# 3D printing of recombinant collagen/chitosan methacrylate/nanoclay hydrogels loaded with Kartogenin nanoparticles for cartilage regeneration

**DOI:** 10.1093/rb/rbae097

**Published:** 2024-08-28

**Authors:** Wanting Zhang, Kejia Shi, Jianfeng Yang, Wenjing Li, Yang Yu, Yu Mi, Tianyu Yao, Pei Ma, Daidi Fan

**Affiliations:** Engineering Research Center of Western Resource Innovation Medicine Green Manufacturing, Ministry of Education, School of Chemical Engineering, Northwest University, Xi’an 710069, China; Shaanxi Key Laboratory of Degradable Biomedical Materials and Shaanxi R&D Center of Biomaterials and Fermentation Engineering, School of Chemical Engineering, Northwest University, Xi’an 710069, China; Biotech. & Biomed. Research Institute, Northwest University, Xi’an 710069, China; Engineering Research Center of Western Resource Innovation Medicine Green Manufacturing, Ministry of Education, School of Chemical Engineering, Northwest University, Xi’an 710069, China; Shaanxi Key Laboratory of Degradable Biomedical Materials and Shaanxi R&D Center of Biomaterials and Fermentation Engineering, School of Chemical Engineering, Northwest University, Xi’an 710069, China; Biotech. & Biomed. Research Institute, Northwest University, Xi’an 710069, China; Engineering Research Center of Western Resource Innovation Medicine Green Manufacturing, Ministry of Education, School of Chemical Engineering, Northwest University, Xi’an 710069, China; Shaanxi Key Laboratory of Degradable Biomedical Materials and Shaanxi R&D Center of Biomaterials and Fermentation Engineering, School of Chemical Engineering, Northwest University, Xi’an 710069, China; Biotech. & Biomed. Research Institute, Northwest University, Xi’an 710069, China; Engineering Research Center of Western Resource Innovation Medicine Green Manufacturing, Ministry of Education, School of Chemical Engineering, Northwest University, Xi’an 710069, China; Shaanxi Key Laboratory of Degradable Biomedical Materials and Shaanxi R&D Center of Biomaterials and Fermentation Engineering, School of Chemical Engineering, Northwest University, Xi’an 710069, China; Biotech. & Biomed. Research Institute, Northwest University, Xi’an 710069, China; Engineering Research Center of Western Resource Innovation Medicine Green Manufacturing, Ministry of Education, School of Chemical Engineering, Northwest University, Xi’an 710069, China; Shaanxi Key Laboratory of Degradable Biomedical Materials and Shaanxi R&D Center of Biomaterials and Fermentation Engineering, School of Chemical Engineering, Northwest University, Xi’an 710069, China; Biotech. & Biomed. Research Institute, Northwest University, Xi’an 710069, China; Engineering Research Center of Western Resource Innovation Medicine Green Manufacturing, Ministry of Education, School of Chemical Engineering, Northwest University, Xi’an 710069, China; Shaanxi Key Laboratory of Degradable Biomedical Materials and Shaanxi R&D Center of Biomaterials and Fermentation Engineering, School of Chemical Engineering, Northwest University, Xi’an 710069, China; Biotech. & Biomed. Research Institute, Northwest University, Xi’an 710069, China; Engineering Research Center of Western Resource Innovation Medicine Green Manufacturing, Ministry of Education, School of Chemical Engineering, Northwest University, Xi’an 710069, China; Shaanxi Key Laboratory of Degradable Biomedical Materials and Shaanxi R&D Center of Biomaterials and Fermentation Engineering, School of Chemical Engineering, Northwest University, Xi’an 710069, China; Biotech. & Biomed. Research Institute, Northwest University, Xi’an 710069, China; Engineering Research Center of Western Resource Innovation Medicine Green Manufacturing, Ministry of Education, School of Chemical Engineering, Northwest University, Xi’an 710069, China; Shaanxi Key Laboratory of Degradable Biomedical Materials and Shaanxi R&D Center of Biomaterials and Fermentation Engineering, School of Chemical Engineering, Northwest University, Xi’an 710069, China; Biotech. & Biomed. Research Institute, Northwest University, Xi’an 710069, China; Engineering Research Center of Western Resource Innovation Medicine Green Manufacturing, Ministry of Education, School of Chemical Engineering, Northwest University, Xi’an 710069, China; Shaanxi Key Laboratory of Degradable Biomedical Materials and Shaanxi R&D Center of Biomaterials and Fermentation Engineering, School of Chemical Engineering, Northwest University, Xi’an 710069, China; Biotech. & Biomed. Research Institute, Northwest University, Xi’an 710069, China

**Keywords:** cartilage regeneration, recombinant collagen, Kartogenin, 3D printing

## Abstract

Cartilage defects are frequently caused by trauma, illness and degradation of the cartilage. If these defects are not sufficiently treated, the joints will degrade irreversibly, possibly resulting in disability. Articular cartilage lacks blood vessels and nerves and is unable to regenerate itself, so the repair of cartilage defects is extremely challenging in clinical treatment. Tissue engineering technology is an emerging technology in cartilage repair and cartilage regeneration. 3D-printed hydrogels show great potential in cartilage tissue engineering for the fabrication of 3D cell culture scaffolds to mimic extracellular matrix. In this study, we construct a 3D-printed hydrogel loaded with nanoparticles by electrostatic interaction and photo cross-linking for the regeneration of cartilage, which has adaptable and drug-continuous release behavior. A photopolymerizable bioink was prepared using recombinant collagen, chitosan, nanoclay Laponite-XLG and nanoparticles loaded with Kartogenin (KGN). This bioink was added with KGN, a small molecule drug that promotes cartilage differentiation, and as a result, the 3D-printed CF/CM/3%LAP/KGN scaffolds obtained by extrusion printing is expected to be used for cartilage repair. It was shown that the 3D-printed scaffolds had good cytocompatibility for human bone marrow mesenchymal stem cells (hBMSCs) and exhibited excellent antimicrobial properties, the continuous release of KGN in the scaffold induced the hBMSCs differentiation into chondrocytes, which significantly enhanced the expression of collagen II and glycosaminoglycan. *In vivo* studies have shown that implantation of KGN-loaded scaffolds into cartilage-injured tissues promoted cartilage tissue regeneration. This study demonstrated that 3D-printed CF/CM/3%LAP/KGN scaffolds can be used for cartilage repair, which is expected to lead to new healing opportunities for cartilage injury-based diseases.

## Graphical Abstract

**Figure:**
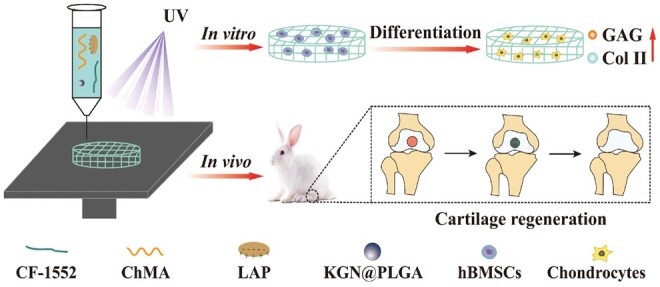

## Introduction

In recent years, the increasing number of patients with cartilage defects has become a globally prevalent condition that places a heavy financial strain on both healthcare systems and patients. Current treatments, such as microfracture repair, onlay plasty, autologous and allogeneic osteochondral regeneration, as well as chondrocyte regeneration, can alleviate pain and enhance joint function [1]. However, the majority of these treatments encounter challenges in achieving durable integration with surrounding host tissues. Consequently, fibrocartilage formation often occurs, compromising the composition and function of the regenerated cartilage, and potentially exacerbating complications [2]. Each treatments have its own limitations, which does not meet the requirements of clinic.

Tissue engineering technology is an emerging technology in cartilage repair and cartilage regeneration, with an important goal of creating biomimetic extracellular matrix (ECM) biomaterials. The structure of hydrogels is very similar to the ECM of mammalian tissues, which shows great potential to be an appropriate material for cartilage regeneration. 3D printing is a method of preparing hydrogels [3]. This technology can reconstruct the shape and function of the injured area. Its rapid prototyping characteristics make it suitable for personalized treatments and enable precise control over the target structure [4]. 3D printing is widely used in tissue engineering and regenerative medicine compared to other technologies. Furthermore, 3D printing technology enables the delivery of cells, growth factors or nanoparticles to the site of injury for therapeutic purposes, thereby promoting cartilage regeneration [5].

Collagen is the primary component of chondrocyte ECM and plays an important role in cartilage regeneration as it has been reported to promote cell adhesion and proliferation [6]. Recombinant collagen CF-1552 (CF-1552) is the mRNA of human collagen reverse transcribed to generate cDNA, which is then expressed in *Escherichia coli* BL21 [7, 8]. Recombinant collagen has the potential to be a promising alternative to animal collagen due to its good biocompatibility, biodegradability and especially low risk of infections and immunological reactions [9]. Several studies have been reported on the use of CF-1552 for cartilage regeneration [10, 11]. However, due to its lack of mechanical strength, the application of CF-1552 in biomedicine is still substantially limited and has not yet been applied to 3D printing technology.

Chitosan is a biodegradable natural biomaterial containing a large number of amino and carboxyl groups, which has a structure similar to glycosaminoglycans (GAG, a component of ECM) and facilitates cell adhesion [12]. Chitosan has a wide range of applications in 3D printing due to its favorable properties [13], including biodegradability, non-toxicity, biocompatibility, antimicrobial properties [14, 15]. Chitosan can be chemically modified by methacryloyl groups to produce the water-soluble, photocrosslinkable polymer chitosan methacrylate (ChMA). Combining ChMA with CF-1552, which would address the poor mechanical strength of CF-1552, in addition, this protein-polysaccharide structure could provide an ECM-like environment for chondrocyte growth, which would be promising to promote cartilage regeneration.

LAPONITE^®^ XLG nanosilicate (LAP, ${\text{Na}}_{0.7}^{+}$[(Mg_5.5_Li_0.3_)${\text{Si}}_{8}^{-}$O_20_(OH)_4_]_0.7_^−^) is a disc-shaped 2D nanoclay particle with a thickness of 1–2 nm, which has positively charged edges and negatively charged surfaces [16]. This unique structure allows LAP to interact electrostatically with charged components [17]. Therefore, chitosan with a positive charge can interact with the negatively charged surface of LAP through electrostatic interactions, leading to binding between them [18]. Furthermore, LAP is an FDA-approved material that is generally regarded as safe for biomedical application [19]. Researchers often incorporate LAP as a rheology modifier into bioinks [19, 20], thereby improving the printability of bioinks [17].

Johnson *et al.* [21, 22] made the initial discovery of the small molecular chemical known as Kartogenin (KGN). By modifying the CBF-β/Runx1 signaling pathway, it promotes the differentiation of mesenchymal stem cells (MSCs) into cartilage and shields it from damage [23, 24]. However, the hydrophobicity of KGN limits its application. In order to achieve drug release, polymers have been widely employed as drug carriers in drug delivery [25–27]. Solvent evaporation is one of the most commonly used methods to prepare drug-loaded polymer particles, which prevents drug degradation during encapsulation and thus increases the drug-loading capacity [28]. It has been shown that the incorporation of nanoparticles encapsulated with KGN in scaffolds can enable targeted drug delivery and thus promote cartilage regeneration [29, 30].

In this study, we fabricated a recombinant collagen-based hydrogel scaffold using 3D printing technology to provide a biomimetic cartilage microenvironment, doping LAP into the hydrogel to improve the printability of the material, and modifying chitosan by methacrylic anhydride (MA) to provide light-curing properties, thereby enhancing the mechanical properties of the bioink. Meanwhile, in order to further meet the demand for cartilage repair materials, the polymer material PLGA encapsulated KGN was used to prepare nanoparticles. These nanoparticles were then incorporated into the scaffolds to make them capable of inducing chondrocyte differentiation. Our recombinant collagen-based 3D-printed scaffold exhibited similarities to cartilage ECM and exhibited sustained release of KGN. The fabricated 3D-printed scaffold displayed excellent biocompatibility and promoted human bone marrow-derived stem cells (hBMSCs) proliferation. Additionally, it exhibited remarkable antibacterial properties and the ability to induce hBMSCs differentiation into chondrocytes. Furthermore, in a rabbit cartilage defect model, the scaffold demonstrated outstanding efficacy in promoting cartilage defect repair. This work brings new hope for the treatment of cartilage defects and holds significant research significance in the field of cartilage regeneration.

## Experimental

### Materials

Chitosan (≥95% deacetylated) was bought from Aladdin (Shanghai, China). LAPONITE^®^ XLG was purchased from BYK-Chemie GmbH (Wesel, Germany). Recombinant collagen CF-1552 (China patent number: ZL01106757.8, Mr ≈ 97 000) was purchased from Xi’an JUZI Biology Gene Technology Co., Ltd MA (94%) and KGN were obtained from Macklin (Shanghai, China). Polyvinyl alcohol (PVA, Mw = 22 000 g/mol, 88% hydrolyzed) was purchased from Sigma–Aldrich. PLGA (poly(D, L-lactide-co-glycolide)) with Mw = 38 000–54 000 g/mol was bought from Macklin (Shanghai, China).

### Synthesis of ChMA

ChMA was obtained by modifying chitosan using methacrylated anhydride in the following experimental steps [31]: 1 g (0.0062 mol) of chitosan powder was dissolved in 2% (v/v) aqueous acetic acid solution and stirred for 12 h at room temperature to obtain chitosan solution (2 wt%). Then, 1.965 ml (0.0124 mol) of MA was added dropwise to the prepared chitosan solution, heated in a water bath and stirred at 60°C for 3 h, where the ratio of MA to the amino group on chitosan was 1:2. The mixture was dialyzed with ultrapure water for 7 days to remove unreacted MA. The final reaction solution was frozen at −80°C and then lyophilized for two days, and the final product was collected as ChMA.

### Characterization of ChMA

^1^HNMR spectra were obtained using a 400-MHz NMR spectrometer (Bruker Avance NEO 400 MHz, Germany). The samples were scanned 256 times at room temperature with D_2_O (0.4M CH_3_COOH added) as solvent at a concentration of 5 mg/ml. On the ^1^HNMR spectrum, the degree of substitution (DS) of ChMA was determined using Equation (1).

(1) $$\mathrm{DS}=\frac{A_{5.7\mathrm{pppm}\&5.4p\mathrm{pm}}/2}{A_{3.0p\mathrm{pm}\&3.4-3.8p\mathrm{pm}}/6}$$

$A_{5.7\mathrm{pppm}\&5.4p\mathrm{pm}}$ was the integrated area of the methylene proton peaks at 5.7 and 5.4 ppm, and $A_{3.0p\mathrm{pm}\&3.4\;-\;3.8p\mathrm{pm}}$ was the integral area of the cyclic proton peaks of monosaccharides glucosamine and monosaccharides acetylglucosamine, chemically shifted at 3.0 and 3.4–3.8 ppm, respectively.

The infrared spectrum was obtained by Fourier transform infrared spectroscopy (IRAffinity-1S, Shimazu, Japan), and the measurement range was 400–4000 cm^−1^.

### Preparation of KNG@PLGA nanoparticles

In this study, Oil-in-water emulsified solvent volatilization was used to prepare KGN@PLGA nanoparticles [29]. About 30 mg/ml of PLGA and 10 mg/ml of KGN were mixed after being dissolved in dichloromethane and dimethyl sulfoxide, respectively. After that, the mixture was added to 3 ml of PVA aqueous solution (1% w/v), mixed well and then sonicated (40 W) in an ice bath for 6 min to obtain the emulsion. The emulsion was then added dropwise to 60 ml of aqueous PVA solution (0.1% w/v) and stirred at 300 rpm for 12 h at room temperature, thereby evaporating the organic solvent. Afterward, the solution was centrifuged (12 000 rpm, 20 min) to collect the precipitated product and washed five times with ultrapure water, frozen and then lyophilized for two days to collect the KGN@PLGA nanoparticles.

### Characters of KGN@PLGA nanoparticles

#### Nanoparticles morphology

The microstructure of the nanoparticles was examined using a scanning electron microscope (SEM, Carl Zeiss, Germany). The average particle size of the nanoparticles was determined by analyzing 50 individual particles using Image J software (Version 1.53), which calculated their mean size value.

#### Loading and encapsulation rates of nanoparticles

In order to determine the content of KGN in KGN@PLGA nanoparticles, high-performance liquid chromatography (HPLC, Shimazu, Japan) was employed following the addition of 0.1M sodium hydroxide solution to the nanoparticles for their degradation. KGN standard curves were obtained by testing the chromatographic profiles of KGN solutions at concentrations of 1000, 500, 250, 125 and 62.5 μg/ml with methanol as solvent. The test conditions were as follows: the mobile phase was methanol: water (55:45) at a flow rate of 1 ml/min, the detection wavelength was 280 nm, the column temperature was 30°C and the injection volume was 10 μl. The encapsulation (ER) and drug loading (LR) rates were calculated according to the following equations:

$$\begin{array}{c} ER\left(\%\right)=\frac{\mathrm{Weight}\;\mathrm{of}\;\mathrm{encapsulated}\;\mathrm{KGN}\;\mathrm{in}\;\mathrm{nanoparticles}}{\mathrm{Weight}\;\mathrm{of}\;\mathrm{initial}\;\mathrm{KGN}}\times 100\%.\\ LR\left(\%\right)=\frac{\mathrm{Weight}\;\mathrm{of}\;\mathrm{encapsulated}\;\mathrm{KGN}\;\mathrm{in}\;\mathrm{nanoparticles}}{\mathrm{Weight}\;\mathrm{of}\;\mathrm{nanoparticles}}\times 100\%. \end{array}$$

#### Study of KGN release from nanoparticles

The release properties of KGN@PLGA nanoparticles (in a solution containing 10 μM KGN, *n* = 3) were assessed by monitoring the KGN concentration in 1 ml PBS over a duration of 21 days using HPLC analysis. In addition, the release profile of KGN from hydrogel scaffolds was investigated using dialysis method. The hydrogel doped with KGN@PLGA nanoparticles (KGN doping concentration of 10 μM) was placed in a dialysis bag, which was then placed in 100 ml of PBS and incubated in an oscillating incubator for 21 days. Samples were taken at regular intervals and replenished with the same volume of fresh PBS. The concentration of KGN released alone and in hydrogels was determined at 280 nm.

### Fabrication of the printable nanocomposite bioinks

According to reports, KGN has been shown to effectively promote stem cell regeneration at a concentration of 100 nM [22]. Dong *et al.* [32] investigated the release behavior of PLGA-loaded KGN at concentrations of 1, 10 and 100 μM, as well as the mechanical properties of the hydrogel. They found that when the loading concentration was 10 μM, the released KGN from the hydrogel exhibited good bioactivity. Therefore, in this study, a concentration of 10 μM of loaded KGN was used with the expectation of preserving its favorable biological functionality.

About 10% w/v CF-1552, 1% w/v ChMA and 0.2% w/v lithium phenyl (2,4,6-trimethylbenzoyl) phosphonate (Mackin, Shanghai, China) were added to deionized water. Then, the deionized water was quickly added to the LAP powder and rapidly stirred at room temperature (500 rpm) for 1 h to prepare 4% w/v, 6% w/v or 8% w/v LAP solutions, respectively. KGN@PLGA nanoparticles were added to deionized water and sonicated for 1 h to obtain a uniformly dispersed suspension of KGN@PLGA nanoparticles, which was then added to the LAP gel solution and stirred for 4 h to load the KGN@PLGA into the LAP solution. Finally, the two mixtures prepared above were mixed in equal volume proportions to obtain a homogeneous pregel solution containing 10 μM KGN, which was used as the bioink for next 3D printing step. The concentrations of nanocomposite bioinks used for 3D printing are shown in Table 1.

**Table 1.** The concentrations of the nanocomposite bioinks

	CF-1552 (w/v %)	ChMA (w/v %)	LAP (w/v %)	KGN (μM)
2%LAP	5	0.5	2	10
3%LAP	5	0.5	3	10
4%LAP	5	0.5	4	10

### Extrusion-based 3D printing

STL files were generated from Autodesk Thinkcad tool. Cura (Cura 15.02.1) was used to generate the G-code based on STL files. A 3D bioprinter (PC Printer, Particle Cloud Biotechnology, Xi’an, China) was used for the fabrication of constructed objects. The cylinder was loaded with three composite bioinks containing different concentrations of LAP (CF/CM/2% LAP/KGN, CF/CM/3% LAP/KGN and CF/CM/4% LAP/KGN bioinks), and a stainless-steel needle with a gauge of 25G (260 μm inner diameter) was used to extrude the loaded bioink. The printing speed was 30 mm/s, the printing temperature was 25°C and the extrusion rate was 5%. While printing, the printed structures on the platform were exposed to a UV light source (365 nm, 10 mW/cm^2^) for photo-cross-linking of hydrogel.

## Characterization

### Rheological measurements

The rheological properties of CF/CM/3%LAP, CF/CM/4%LAP, CF/CM/3%LAP/KGN and CF/CM/4%LAP/KGN bioinks were characterized using a rotational rheometer (MCR-92 Anton Paar, USA). The extrudability of the unphotocrosslinked bioinks was verified using steady-state rate scans (0.1–1000 s^−1^). Next, a constant strain of 1% was set and the hydrogels after photocrosslinking were used to verify the stability of the hydrogels using an oscillatory frequency scan (0.1–100 Hz).

### Uniaxial compressive testing

Cylindrical hydrogel specimens (*n* = 3) underwent unconfined compression testing utilizing an Instron universal testing machine (WDW 3020, USA) with a crosshead speed of 5.0 mm/min. To safeguard the load cell, the compression limit was established at a maximum strain of 70%. The Young’s modulus was determined by analyzing the slope of the stress–strain curve.

### *In vitro* biocompatibility testing

The hBMSCs were obtained from Shanghai Fu Heng Biology Co., Ltd (Shanghai, China). The hBMSCs were cultured in a low-glucose Dulbecco’s modified Eagle’s medium (DMEM, Gibco), which was supplemented with 10% fetal bovine serum (FBS, Gibco) and 1% penicillin/streptomycin (Gibco). The cells were passaged upon almost 80% confluence and only hBMSCs prior to six-passage were used for experimental studies. Leaching liquors were collected from the printed composite hydrogels that had been immersed in cell medium at 37°C for 3 days. hBMSCs were seeded into 96-well plates and incubated with the leaching liquor. After 24 or 48 h of cell culture, each well was supplemented with 50 μl of MTT (Solarbio, China) solution (5 mg/ml), and the cells were cultured for 2–4 h. The original culture solution was sucked away, and 150 μl of DMSO was added to each well, and the OD value was measured by an ELISA (Biotek, USA) at 490 nm (*n* = 3). Meanwhile, for cells in well plates incubated for 24 h, live-dead staining was performed using the AO/EB viability assay kit (Leagene, China). Briefly, the original medium was aspirated, washed with PBS and 100 μl of staining solution was added to the well plates, which were then returned to the incubator and incubated for 30 min. The cells were observed and captured using a fluorescent microscope (Nikon, Japan).

### *In vitro* cell proliferation assay

The cell proliferation capability of the hydrogels was assessed using the BeyoClick™ EdU Cell Proliferation Kit with TMB (Beyotime Institute of Biotechnology, Shanghai, China). In brief, hBMSCs were seeded at a density of 5000 cells per well in a 96-well plate, and cells were cultured with the extraction solutions from different hydrogels for 1 day, 3 days and 5 days, according to the kit instructions (*n* = 3). The absorbance values were recorded at 370 nm using an ELISA reader (Biotek, USA).

Furthermore, Ki67 staining was performed to further confirm the cell proliferation capability of the hydrogels. Glass coverslips were washed with 75% ethanol and air-dried, and then placed in a 6-well plate. Each well was inoculated with hBMSCs cell suspension (1 × 10^4^ cells per well). After allowing the cells to grow well, they were cultured with the extraction solutions from different hydrogels for 3 days. Subsequently, the cells were washed twice with PBS, fixed with 1 ml of 4% paraformaldehyde for 15 min and the fixative was aspirated. Following this, the cells were washed three times with PBS. A circle was drawn on the coverslip using a hydrophobic marker pen, and then 1 ml of 3% BSA was used to block the coverslip at room temperature for 30 min. The blocking solution was aspirated, and the coverslip was incubated overnight at 4°C with Ki67 primary antibody (GB151499, Servicebio, China). After incubation, the primary antibody was aspirated, and the cells were washed three times with PBS. Next, CY3-conjugated goat anti-rabbit IgG secondary antibody (GB21303, Servicebio, China) was added to the coverslip and incubated at room temperature for 50 min. The secondary antibody was aspirated, and the cells were washed three times with PBS. Finally, the cells were stained with DAPI staining solution and incubated at room temperature for 10 min in the dark. After washing three times with PBS, the coverslip was mounted using an anti-fluorescence quenching mounting medium. Immunofluorescence data were collected using a scanner (Pannoramic MIDI, 3DHISTECH), and the stained images were obtained using CaseViewer 2.4.

### Chondrogenic differentiation of hBMSCs on 3D-printed scaffolds

Scaffolds were 3D-printed from CF/CM/3%LAP and CF/CM/3%LAP/KGN bioinks as described in the section ‘Extrusion-based 3D printing’. Scaffolds implanted with hBMSCs were stained using Vari Fluor 555-Phalloidin (MCE, USA) and 4′,6-diamidino-2-phenylindole (Solarbio, China) to assess the scaffold attachment effect on cells. Detailed information regarding the aforementioned experimental steps can be found in the electronic Supplementary Material.

hBMSCs were subjected to chondrogenic differentiation to evaluate the regenerative potential of each scaffold for articular cartilage. hBMSCs were seeded at a density of 2 × 10^5^ cells/well on each scaffold in 24-well cell culture well plates, and cultured with the DMEM medium for 24 h. Subsequently, the medium was replaced with chondrogenic differentiation medium (CTCC-Y002; CTCC Biosciences Inc., Jiangyin, China) and continuously cultured for 3 weeks. hBMCSs co-cultured without the addition of scaffolds were set as control groups, in which negative control cells were continuously cultured with DMEM medium for 3 weeks and positive control cells were continuously cultured with chondrogenic differentiation medium for 3 weeks. After incubation for 21 days, the chondrogenesis was evaluated through histochemical staining using the toluidine blue assay. Briefly, the scaffolds were rinsed with PBS, fixed with 4% paraformaldehyde for 20 min and then rinsed with PBS. The differentiated cells on each scaffold were subjected to staining using a solution of toluidine blue (for cells, Solarbio, China). The scaffolds were fully rinsed in PBS to remove excess dye before imaging under a microscope.

The cell culture steps for biochemical index analysis are the same as above. Collagen II (Col II) and GAG measurements were performed on hBMSCs after 7, 14 and 21 days of chondrogenic differentiation medium induction (control) and scaffolds induction. Samples (*n* = 3) were initially rinsed with PBS and subsequently transferred to 1.5 ml Eppendorf tubes. Each sample underwent three freeze/thaw cycles, alternating between −20°C and 37°C. Following the freeze/thaw cycles, the lysate was collected from each sample and utilized for quantifying the amount of GAG and Col II. Col II was assessed using a type II collagen ELISA kit (TSZ ELISA, USA) following the guidelines provided by the manufacturer. Briefly, Standard and sample wells are added and horseradish peroxidase-labeled detection antibody is added to each well and incubated for 60 min. After removing the antibody and rinsing the plate, the substrate was added and incubated for 15 min. Finally, each well was mixed with a termination solution and the OD value was measured at 450 nm. The GAG concentration was assessed through spectrophotometric analysis by adding 25 μl of lysate, 5 μl of NaCl solution (2.3 M) and 150 μl of dimethylmethylene blue dye (DMMB, Sigma Aldrich) to the lysate solution [33]. The absorbance at 525 nm was promptly measured using an ELISA (Biotek, USA) to quantify the intensity of the color change, enabling the determination of GAG concentration.

### *In vivo* cartilage regeneration assessment

#### Animals and surgical protocol

All animal procedures were approved by the Animal Ethics Committee of Northwestern University (NWU-AWC-20220902L), which included the following: mature male New Zealand Large White rabbits (2.5–3 kg, *n* = 4/time point/group) were selected. The knees of the rabbits were divided into three groups: the control group (treated with sutures and injections of penicillin sodium only), the CF/CM/3% LAP scaffolds group and CF/CM/3% LAP/KGN scaffolds group. The rabbit’s knee joint was immobilized, and an incision was made on the lateral side of the rabbit’s patella with a scalpel to expose the articular cartilage. Subsequently, a full-thickness cartilage defect (2.5 mm in diameter and 1.5 mm in depth) in the rabbit’s articular cartilage was created using a dental drill. The defect was then filled with a printed hydrogel scaffold for treatment, and the control group was not filled with a hydrogel scaffold. After suturing the skin layer by layer, the skin wound was sterilized using iodophor. Sodium penicillin was injected intramuscularly into all rabbits 3 days after surgery to prevent infection, and the knee joints were collected and evaluated at 6 and 12 weeks after surgery.

#### Micro-computed tomography (micro-CT) measurement

The samples were fixed in 4% paraformaldehyde overnight and then evaluated for their morphologies using a micro-CT scanner (SkyScan 1276, Bruker, Germany). The scanning parameters were set as follows: voxel size of 10 μm, 70 kV, 200 μA, 1 mm Al filter and integration time of 350 ms. NRecon (NRecon 1.7.4.2) was utilized to analyze the reconstructed micro-CT images. CTvox (CTvox 3.3) was employed to generate 3D images from contoured 2D images. DataViewer (DataViewer 1.5.1.9) was used to extract 2D images of specific areas of interest from the 3D images. CTAn (CTAn 1.20.3) was utilized to calculate the bone volume fraction (BV), bone total volume (TV), bone trabecular thickness (Tb.Th) and bone mineral density (BMD) for evaluating indicators of subchondral bone reconstruction.

#### Histological

The collected kneecap tissue was subjected to decalcification, embedding and sectioning processes, and all staining procedures were conducted on tissue sections with a thickness of 3–4 μm. Hematoxylin and Eosin (H&E), Masson’s trichrome, toluidine blue staining, Safranin O/Fast Green staining and Col II immunohistochemical staining were performed following the instructions provided by the respective manufacturers. Subsequently, images were captured using a low-magnification light microscope (Nikon, Japan).

### Statistical analysis

Data analysis and graph processing were performed using GraphPad Prism 8.0.2 and ImageJ. The experimental data were analyzed using t-tests, two-way ANOVA or one-way ANOVA and presented as mean±SD. A significance level of *P* < 0.05 was used to determine statistical differences.

## Results and discussion

### Synthesis and characterization of ChMA

**Figure 1.**
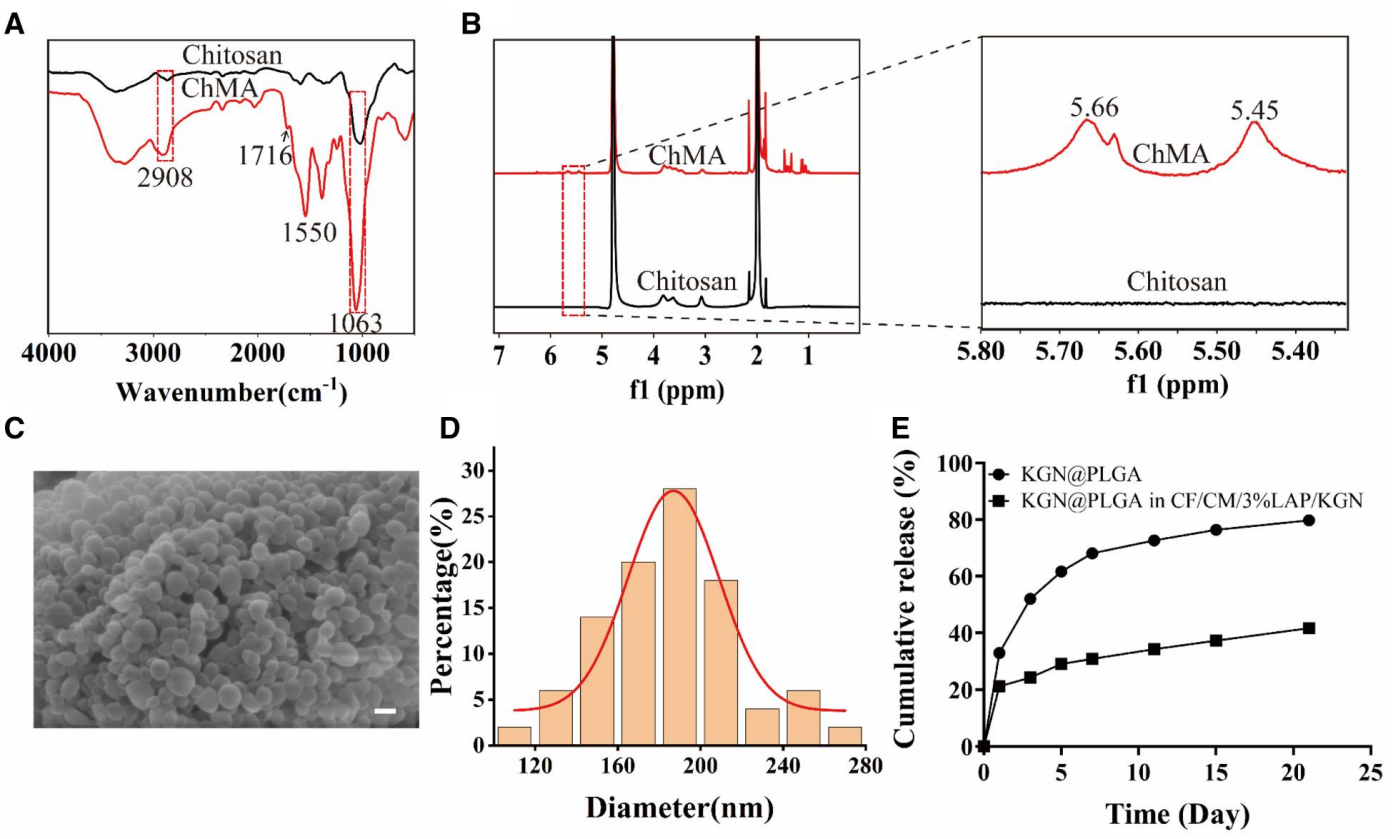
(**A**) FTIR spectra of chitosan and ChMA. (**B**) ^1^H NMR spectra of chitosan and ChMA. (**C**) SEM image of KGN@PLGA nanoparticles. (**D**) Particle size analysis of KGN@PLGA nanoparticles. (**E**) Cumulative release of KGN from KGN@PLGA nanoparticles and CF/CM/3%LAP/KGN scaffolds.

Chitosan is considered as an outstanding substrate material for bone tissue engineering. However, improvements are highly needed due to its inherent physical and chemical limitations. In our study, we prepared ChMA by the reaction of chitosan with MA. ChMA was characterized using FTIR and NMR. As shown in Figure 1A, the characteristic absorption bands for chitosan were observed at 1063 cm^−1^(amine C–N stretch). The increased intensity of absorption bands at 2908 cm^−1^ indicated the presence of ChMA, as both chitosan and methacrylate group exhibited alkyl C–H stretch. The appearance of a strong signal at 1550 cm^−1^ depicted N–H stretch, while the amide C = O stretch evident at 1716 cm^−1^ in ChMA confirmed the methacrylation of chitosan. As depicted in Figure 1B, the presence of olefin double peaks at 5.45 and 5.66 ppm indicated the successful grafting of MA’s carbon–carbon double bonds onto Chitosan. The DS for ChMA was determined to be 16% according to Equation (1).

### Characterization of nanoparticles

Figure 1C depicted the SEM image of KGN@PLGA nanoparticles, demonstrating a uniform spherical shape characterized by a smooth surface with no visible imperfections or pores. The nanoparticles exhibited an average diameter of 185.05 ± 32.86 nm, as shown in Figure 1D. The ER and LR of KGN@PLGA were determined as 23.5% and 5.9%, respectively, using HPLC analysis. These values indicated the successful incorporation of hydrophobic KGN into the PLGA nanoparticles. The *in vitro* release profiles of KGN from PBS and the CF/CM/3%LAP/KGN scaffolds were investigated, as presented in Figure 1E. The results demonstrated that incorporation of KGN@PLGA into the hydrogel slowed down the release rate of KGN. Compared to the release of KGN in PBS, the release rate in hydrogel on the first day was reduced from 32.95% to 21.20%, which prevented the sudden release of the drug. Furthermore, KGN was continuously released from the hydrogel over a period of 21 days.

### Printability

**Figure 2.**
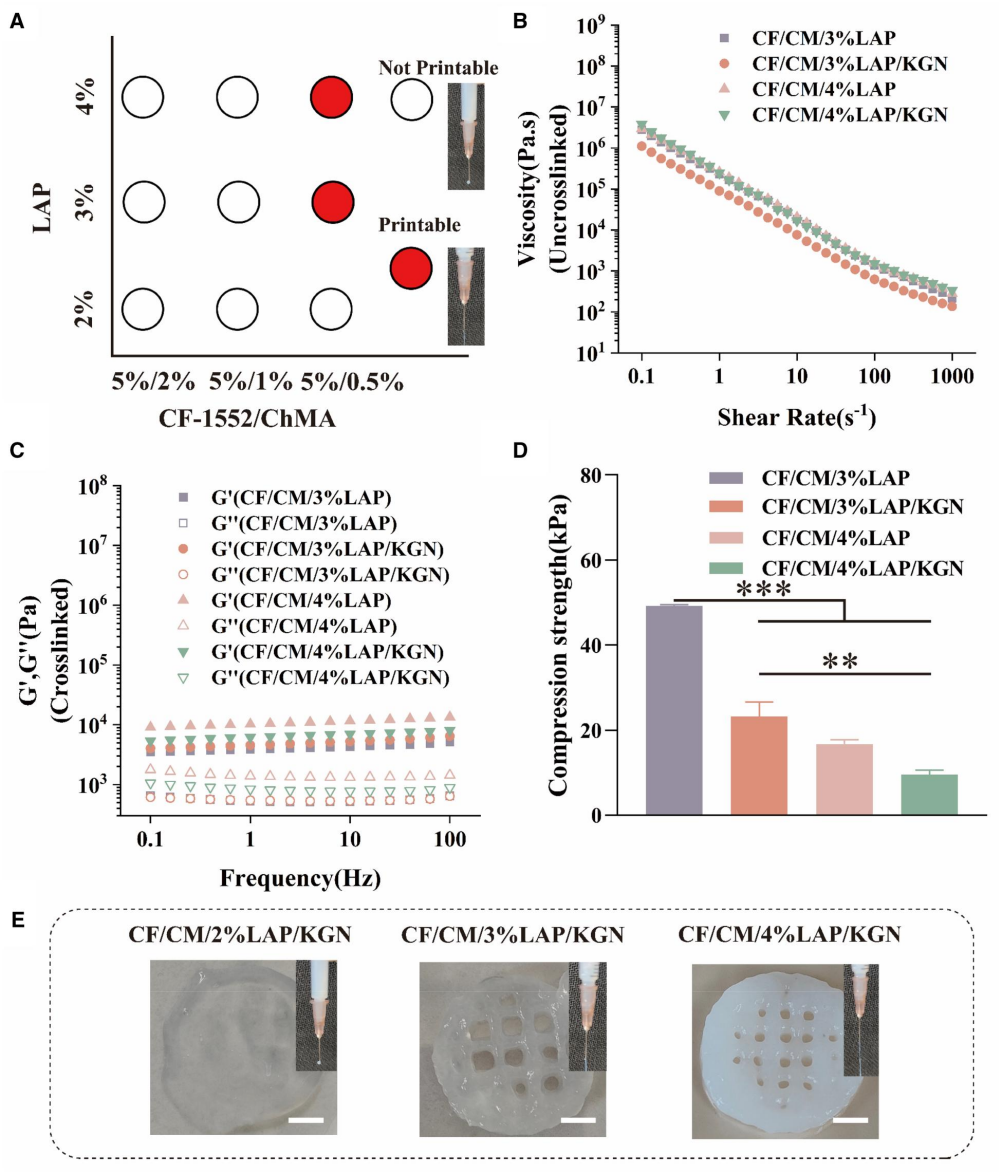
(**A**) Printability of CF1552/ChMA/LAP inks with different concentrations. (**B**) Viscosity-shear rate profile of different bioinks. (**C**) Storage and loss moduli of different bioinks. (**D**) Compressive modulus of different bioinks (**P < 0.01, ***P < 0.001). (**E**) Photographs of the printed constructs from CF/CM/2%LAP/KGN, CF/CM/3%LAP/KGN and CF/CM/4%LAP/KGN scaffolds. Scale bars: 5 mm.

The printability of a biomaterial refers to its ability to enable continuous and smooth flow of ink through the nozzle, allowing for the formation of regular and stable filaments during deposition and solidification [34]. In order to assess the printability of CF/CM/LAP, the concentrations of ChMA and LAP were varied while maintaining a constant CF-1552 content (Figure 2A). It was observed that when the ChMA concentration was 0.5% and the LAP concentration not less than 3%, the formulated biomaterial ink flowed smoothly through the needle and extruded a homogeneous filament, displaying favorable printability. However, when the ChMA concentration surpassed 0.5%, the biomaterial ink became excessively viscous, leading to needle clogging and rendering it unsuitable for bioprinting. Conversely, reducing the ChMA concentration below 0.5% resulted in extremely low viscosity, causing the extruded material to behave as droplets and lose printability. Therefore, for extrusion-based printing and biological evaluations, CF-1552/ChMA/LAP bioink formulations with concentrations of 3 wt% or 4 wt% LAP, 0.5 wt% ChMA and 5 wt% CF-1552 were chosen for further experimentation.

### Rheological properties

To assess the printability of the biomaterial, we investigated the rheological properties of the ink. The results, as shown in Figure 2B, revealed that CF/CM/3%LAP or CF/CM/4%LAP bioink exhibited a shear-thinning behavior when subjected to flow, as evidenced by the decreasing viscosity with increasing shear rate. Especially, when KGN@PLGA nanoparticles were doped into the bioink, the viscosity of the ink was found to be similarly similar to that before doping. This observation indicated that the addition of KGN@PLGA nanoparticles did not significantly affect the shear-thinning nature of the bioink. This could be attributed to the amount of KGN@PLGA nanoparticles doped into the hydrogels was relatively small. Furthermore, the KGN@PLGA nanoparticles could have had good dispersibility and stability, thereby avoiding aggregation or gelation of the bioink and maintaining its shear-thinning properties. Consequently, the incorporation of KGN@PLGA nanoparticles into the bioinks formulation did not compromise the fabrication process. The viscosities of all the nanoparticles-bioink formulations remained sufficiently high, ensuring their suitability for 3D printing.

Moreover, after the ink was photo-crosslinked, we measured the storage modulus and loss modulus of the resulting hydrogels. It was observed, as shown in Figure 2C, that these moduli were less dependent on the oscillation frequency. This finding suggested the formation of robust bonds between the methacrylate groups of various ChMA molecules, indicating successful crosslinking. Importantly, the presence of KGN@PLGA nanoparticles did not hinder the photo-crosslinking process of the inks.

Overall, these findings demonstrated that the biomaterial ink, specifically CF/CM/3%LAP/KGN or CF/CM/4%LAP/KGN bioinks, exhibited favorable rheological properties for 3D printing. The ink maintained its shear-thinning behavior, high viscosity and successful crosslinking capability, and the addition of KGN@PLGA nanoparticles has virtually no effect on the printing process.

### Print fidelity

Based on printability determination and rheological studies, CF-/ChMA/2%LAP/KGN, CF-1552/ChMA/3%LAP/KGN and CF-1552/ChMA/4%LAP/KGN bioinks formulations were chosen for 3D printing. These biomaterial inks were extruded through 3D printers and printed into 3D structures using a photo-crosslinking mechanism with the presence of photoinitiator. As shown in Figure 2E, CF-1552/ChMA/3%LAP/KGN and CF-1552/ChMA/4%LAP/KGN bioinks successfully printed multilayered circular hydrogels with a grid-like pattern (inner diameter 20 mm, height 1 mm). In contrast, the product extruded by CF-1552/ChMA/2%LAP/KGN ink did not exhibit porosity, indicating a failed print. The inclusion of Laponite XLG in the three-component ink facilitated strong interactions with anionic, cationic and neutral polymers, resulting in a physically crosslinked network with shear-thinning properties. This is crucial for maintaining the printability of the bioink, allowing the ink to be printed without structural collapse. The light-curing properties of ChMA enabled rapid gelation of the ink, and subsequent UV crosslinking further enhanced the mechanical integrity of the printed structure.

### Investigation of physical and chemical properties of scaffolds

The network morphology of hydrogels is an important factor in determining the mechanical properties and drug release. As shown in Supplementary Figure S1A, observed from the microstructure, the addition of LAP increased the roughness of the pore wall of the hydrogel, and a uniform pore structure could not be formed when LAP was added at 4%, KGN@PLGA nanomicrospheres and LAP nanosheets could be seen in the CF/CM/3%LAP/KGN hydrogel, and there was no obvious aggregation of nanoparticles in the composite hydrogel. In addition, compared to CF/CM/3%LAP hydrogel, CF/CM/3%LAP/KGN exhibits lower compressive strength. This could be attributed to the impact of incorporating KGN nanoparticles on the degree of photocrosslinking [35]. Furthermore, the embedded nanoparticles may interfere with the physical crosslinking between polymers, leading to a decrease in compressive strength [36]. However, it is worth noting that CF/CM/3%LAP/KGN still maintains an excellent pore structure, which is conducive to promoting cell adhesion and differentiation. As shown in Supplementary Figure S1B–D, the average pore sizes of CF/CM, CF/CM/3% LAP and CF/CM/4% LAP hydrogels were 79.14 ± 16.83 μm, 73.05 ± 27.15 μm and 87.09 ± 33.85 μm, respectively, suggesting that the pore sizes of the hydrogels with the addition of 3 wt% LAP became smaller, which may be due to the electrostatic interactions between LAP and methacrylated chitosan and the hydrogen bonding between the polymers, thus enhancing the structural stability of the hydrogels. With the increase of LAP content up to 4 wt%, the pore size of the hydrogel becomes larger and the porous structure formed becomes inhomogeneous, which may be due to the increase of repulsive effect the polymer by the excessive LAP, which hinders the cross-linking of the polymer and the structure formation. While comparing CF/CM/3%LAP and CF/CM/4%LAP hydrogels, the network structure of CF/CM/3%LAP hydrogels is more dense, which leads to an increase in network rigidity.

The results of the compressive property tests of the hydrogels are shown in Figure 2D, comparing CF/CM/3%LAP and CF/CM/4%LAP, which indicate that CF/CM/3%LAP hydrogels have good mechanical properties, with a significant reduction in compressive strength of 32.54 ± 0.76 kPa in the CF/CM/4%LAP group when compared to the CF/CM/3%LAP group (*P* < 0.001). Pore size is an important factor in controlling mechanical strength because in a matrix with a smaller pore size, the load can be distributed uniformly over the entire surface [34]. As a result, CF/CM/3% LAP hydrogels can resist stresses more easily, showing a higher compressive modulus than CF/CM/4% LAP. Therefore, the hydrogel group with 4% LAP added was not suitable for subsequent experiments. It could not provide proper support and protection for chondrocytes as it could not provide sufficient mechanical support and stability.

Supplementary Figure S2 shows the zeta potential values of different component materials. Due to the positively charged amino groups on chitosan and the negatively charged surface of LAP nanosheets, while recombinant collagen is a neutral polymer. The zeta potential of the 3% LAP solution was more negative compared to the zeta potential of the CF/CM/3% LAP hydrogel. This observation suggests the occurrence of electrostatic interactions among the materials. Furthermore, due to the ionization of carboxyl end groups on PLGA and the adsorption of anions in PVA, KGN@PLGA nanoparticles possess a negative surface charge [30, 37]. Therefore, the hydrogel shows a more negative zeta potential trend upon the addition of KGN@PLGA microspheres.

The swelling rate of the scaffolds was tested to evaluate the fluid absorption ability of the tissue engineering scaffolds (Supplementary Figure S3B). The swelling rate of CF/CM/3%LAP scaffolds was 761.65 ± 34.72 (%), CF/CM/4%LAP scaffolds was 798.26 ± 24.20 (%) and the swelling rate did not change much when the concentration of LAP was changed, while the swelling rate of CF/CM/3%LAP/KGN scaffolds was 826.36 ± 8.03 (%), and the addition of KGN@PLGA nanoparticles had a small effect on the swelling rate. The results showed that the CF/CM/3%LAP/KGN scaffolds have sponge-like properties with fast water absorption, and their swelling rate can reach more than 800%, which can rapidly absorb the fluid in the joint defect site and thus achieve close filling with the damaged tissue site.

In the degradation research (Supplementary Figure S3C), the residual weight of the CF/CM/3%LAP/KGN scaffolds decreased slowly with time, and the residual weight of the hydrogel degraded to 75.09 ± 2.64 (%) after 21 days, and the slow degradation of the scaffold enabled the slow release of KGN over a long period of time, which was more favorable to cartilage repair.

### Nanocomposite hydrogel supports cell viability *in vitro*

In this study, Recombinant collagen CF-1552, ChMA and LAPONITE^®^ XLG (LAP) were utilized as the hydrogel materials. Recombinant collagen offers improved water solubility, lower immunogenicity and carries no viral risk compared to conventional animal collagen [38]. Currently, medical devices such as artificial bone and hemostatic sponges developed using CF-1552 as a raw material have been utilized in clinical practice. ChMA is produced by the reaction between chitosan and MA, which serves as a foundational hydrogel material extensively utilized in the field of biomedicine. Numerous studies have provided evidence of ChMA’s favorable biocompatibility [31, 39, 40]. LAP contains trace amounts of heavy metals with low toxic effects [41]. Moreover, it has received FDA approval and is generally considered safe. However, the degradation products of LAP, such as sodium, magnesium and lithium ions, may exhibit cytotoxicity. Studies have indicated that LAP concentrations below 1 mg/ml are non-toxic to human MSCs [42]. Hence, in this study, it is important to evaluate the impact of additional LAP content on hBMSCs’ viability while ensuring the bioink’s printability and maintaining optimal cell vitality.

**Figure 3.**
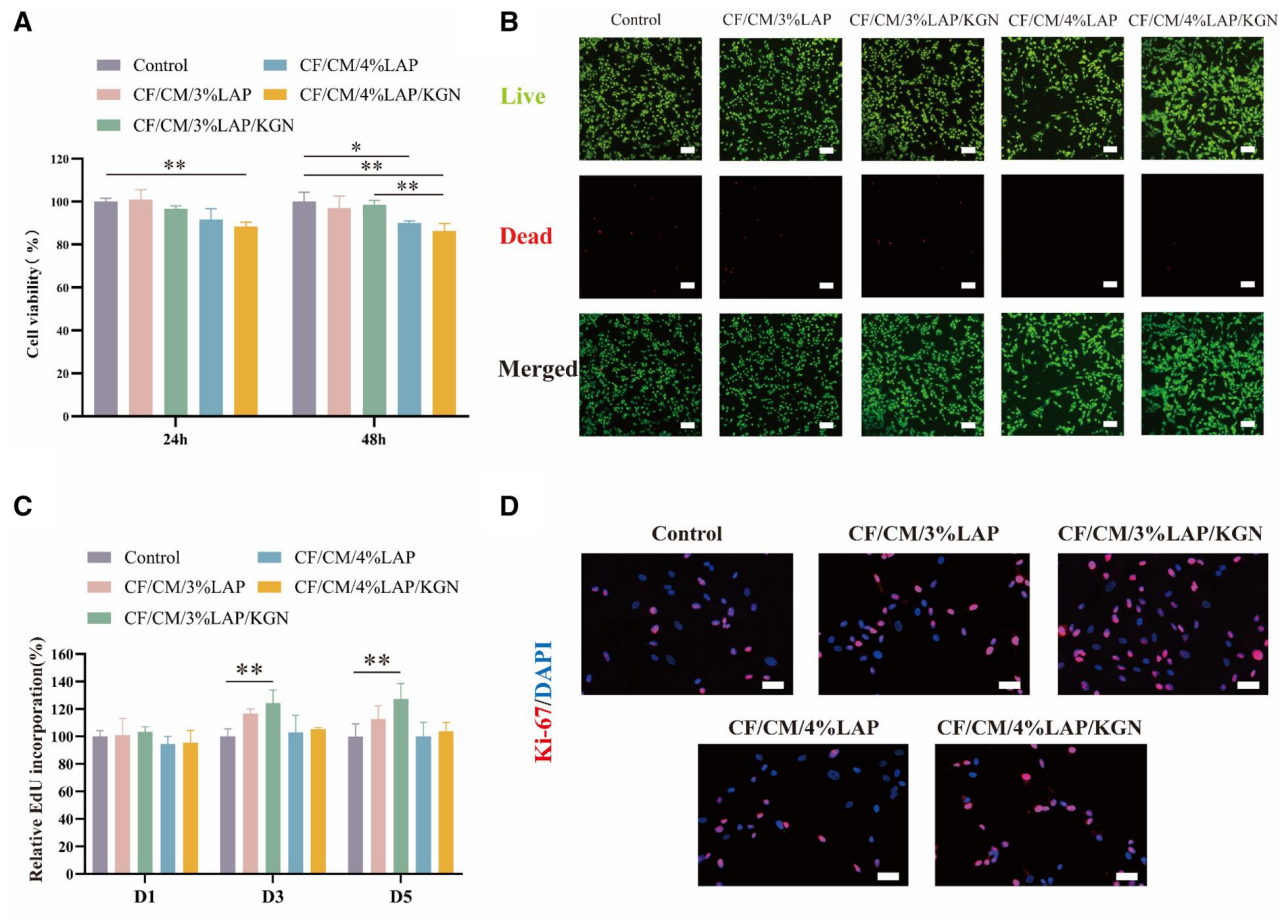
(**A**) Cell viability of hBMSCs in the presence of 3D-printed scaffolds at 24 and 48 h. (**B**) Live/dead staining images to evaluate the hBMSCs viability after 24 h. Scale bar: 200 μm. (**C**) Quantitative evaluation of the ratio of EdU-positive hBMSCs during 5 days of culture in different hydrogels extract solution. (**D**) Immunofluorescent-stained proliferation markers (Ki-67) of hBMSCs after 3 days of culture. Scale bar: 50 μm (*P < 0.05, **P < 0.01).

The cytocompatibility of the hydrogel scaffolds was determined by MTT assay. As shown in Figure 3A, after hBMSCs were co-cultured with the scaffold extract for 24 and 48 h, the cell viability of the CF/CM/3% LAP scaffolds group and CF/CM/3% LAP/KGN scaffolds group reached more than 95%, whereas the cells in the hydrogel group containing 4% LAP exhibited poor cell viability. The results indicated that the hydrogel scaffold doped with 3% LAP exhibited excellent biocompatibility and did not exert significant toxic side effects on hBMSCs (compared to the control, *P* > 0.05).

In addition, live/dead cell (AO/EB) staining was used to analyze the growth of hBMSCs in different material extracts for 24 h. As shown in Figure 3B, no obvious dead cells (red fluorescence) were observed in all materials. This also proved that the scaffolds had good cytocompatibility. In addition, the blood compatibility test (Supplementary Figure S3D) also showed that the hemolysis rate of all the samples in the experimental group was less than 5%, which further proved the biosafety of the prepared scaffold material.

In addition to inducing stem cell differentiation into cartilage, KGN has been demonstrated to promote the proliferation of hBMSCs by activating the AMPK-SIRT1 signaling pathway [43, 44]. In this study, cell proliferation was confirmed through EdU assay. The results demonstrated that the CF/CM/3%LAP/KGN hydrogel exhibited the highest proliferation-promoting ability (Figure 3C). Furthermore, we evaluated the ability of the hydrogel prepared in our study to influence the *in vitro* proliferation of hBMSCs based on immunofluorescence Ki67 staining after a 3-day induction period. The analysis revealed that the majority of cells in the CF/CM/3%LAP/KGN hydrogel exhibited Ki67 positivity, while few Ki67-positive cells were detected in the other treatment groups (Figure 3D), consistent with the conclusions from the EdU assay results. Therefore, these data confirm the ability of the CF/CM/3%LAP/KGN hydrogel to promote the proliferation of BMSCs *in vitro*, highlighting its potential in promoting cartilage regeneration.

### Scaffolds promote cell migration *in vitro*

Due to the physical and chemical properties of the recombinant collagen and chitosan employed in this study, they can effectively mimic the characteristics of the ECM. The ECM, which surrounds cells, plays a pivotal role in cell migration, differentiation and function. The hydrogel incorporating recombinant collagen and chitosan creates a microenvironment that closely resembles the ECM, thereby facilitating directed migration of bone marrow MSCs (BMSCs) and enhancing their recruitment and adhesion [45]. Additionally, previous studies have demonstrated favorable adhesion and proliferation morphology of chondrocytes on CF-1552 hydrogels [38]. Taking advantage of these properties, our scaffold is anticipated to effectively recruit stem cells from the damaged site, ultimately promoting cartilage repair.

The fluorescence microscopy images depicted in Supplementary Figure S4A provide visual evidence of hBMSCs’ behavior on the scaffold, displaying distinct pore wall boundaries and demonstrating the cells’ ability to climb and grow along the scaffold’s pore walls. These observations highlighted the scaffold’s structural integrity and its capability to support cell growth and adhesion. Furthermore, Supplementary Figure S4B, captured by confocal microscopy, allowed the observation of cellular cytoskeleton and nuclei on the scaffold, providing further evidence of the scaffold’s adhesive properties toward hBMSCs. These findings strongly indicate the scaffold’s potential in supporting hBMSCs growth and adhesion.

The results obtained from the scratch test (Supplementary Figure S5) revealed that after 24 h, the migration rates of hBMSCs in the control, CF/CM/3%LAP scaffolds and CF/CM/3%LAP/KGN scaffolds were 21.35 ± 0.82 (%), 29.43 ± 2.41 (%) and 30.67 ± 2.25 (%), respectively. Significantly higher migration rates were observed in the CF/CM/3%LAP/KGN hydrogel scaffolds compared to the control group (*P* < 0.05). These findings suggested that the prepared scaffolds had the ability to recruit stem cells to the defect site, thereby expediting tissue repair.

### Antimicrobial properties of the stent

Prior research has demonstrated that hydrogels based on chitosan exhibit inherent antibacterial properties [46]. When Xi *et al.* [47] added ChMA to the hydrogel, it showed remarkable antibacterial activity against *E.coli* and *Staphylococcus aureus*. This is explained by the hydrogel structure’s amino group, which carries a positive charge. By electrostatic and hydrophobic interactions, chitosan binds to negatively charged bacteria and accumulates on their cell walls. This disruption of the bacterial membrane results in cytoplasmic leaking, which in turn causes bacterial lysis and death [48].

The results of antimicrobial experiments in Supplementary Figure S6 showed that the bacterial survival rate of *E.coli* after co-culture with CF/CM/3%LAP/KGN scaffolds was 5.43 ± 0.98 (%), and that of *S.aureus* was 1.92 ± 0.24 (%), indicating that the scaffolds have excellent antimicrobial properties, which can reduce bacterial infections at the defect site, thus accelerating cartilage repair.

### Chondrogenic differentiation of hBMSCs on scaffolds

Under specific culture conditions, hBMSCs can be induced to differentiate into chondrocytes, leading to the formation of cartilage for tissue regeneration. Toluidine blue is a cationic dye that exhibits high affinity for sulfate groups present in proteoglycans, it can be used to assess the content of proteoglycans secreted during cartilage differentiation [49]. During chondrogenesis, hBMSCs produced fibrous ECM proteins and GAG, with Col II accounting for 90–95% of the collagen produced by chondrocytes in the ECM [50], and GAG is essential for articular cartilage weight-bearing [51]. In this study, a 3-week chondrogenic differentiation experiment was conducted on the CF/CM/3%LAP and CF/CM/3%LAP/KGN scaffolds (Supplementary Figure S7). The hBMSCs induced by DMEM medium without scaffold were the negative control group, and the hBMSCs induced by chondrogenic differentiation medium without scaffold were the positive control group, and the differentiated cells were stained with toluidine blue staining solution after 21 days of culture. Based on the fact that Col II and GAG are specific substrates for cartilage formation, the content of the above two substances secreted by the cells during culture can be used as an evaluation criterion for assessing the pro-chondrogenic effect of the scaffold. The biochemical quantitative analysis of Col II and GAG was performed at predetermined time points of 7, 14 and 21 days.

**Figure 4.**
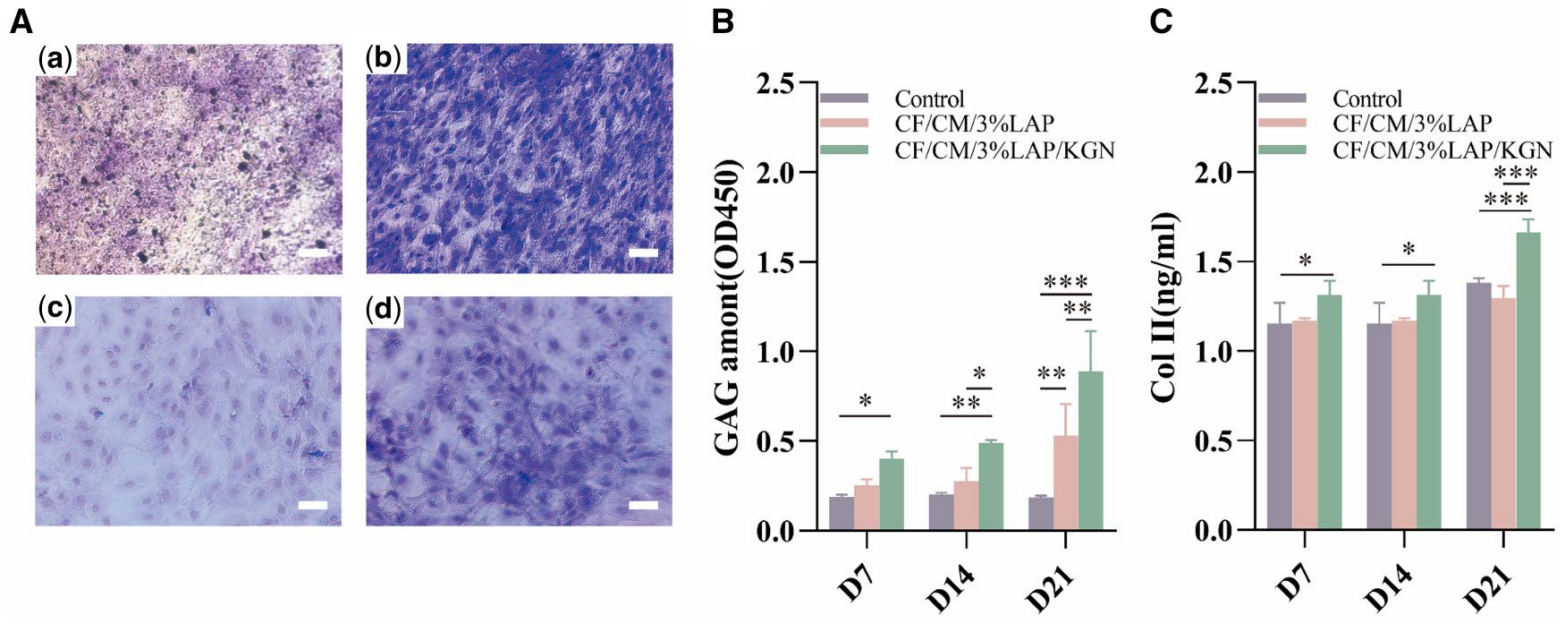
(**A**) Toluidine blue stained micrographs of hBMSCs after 21 days of chondrogenic differentiation on the (**a**) DMEM medium, (**b**) chondrogenic differentiation medium, (**c**) CF/CM/3%LAP scaffolds and (**d**) CF/CM/3%LAP/KGN scaffolds. Scale bar: 200 μm. (**B**) GAG and (**C**) Col II quantification of hBMSCs after 7, 14 and 21 days of chondrogenic differentiation on CF/CM/3%LAP and CF/CM/3%LAP/KGN scaffolds (*P < 0.05, **P < 0.01, ***P < 0.001).

The toluidine blue staining results, as shown in Figure 4A, revealed that the staining intensity in the negative control group cultured in DMEM medium (Figure 4Aa) was significantly weaker compared to the positive control group induced by chondrogenic differentiation medium (Figure 4Ab). This suggested that hBMSCs used in the experiments possess normal differentiation ability into chondrocytes. Furthermore, the positive staining in the CF/CM/3%LAP/KGN scaffolds (Figure 4Ad) was deeper than that in the CF/CM/3%LAP scaffolds (Figure 4Ac) after 21 days, indicating a higher ECM content in the former group. This finding suggested that the scaffold containing KGN promoted the formation of ECM. According to the quantitative analysis results (Figure 4B), the content of GAG in both scaffolds group was significantly increased with time. After 21 days of culture, the GAG amount of the CF/CM/3%LAP/KGN scaffold group was significantly higher than that of the control and CF/CM/3%LAP scaffold groups (*P* < 0.01). The collagen contents in both scaffolds groups also increased gradually with time (Figure 4C), especially the CF/CM/3%LAP/KGN scaffold group showed significantly higher Col II amount than other two groups at Day 21 (*P* < 0.001). This data indicated that the *in vitro* release of KGN from the CF/CM/3%LAP/KGN scaffold effectively promoting the collagen II synthesis and GAG secretion, demonstrating stimulating effects on the chondrogenic differentiation of hBMSCs. Treatment with KGN was found to upregulate the expression of essential chondrogenic markers, including collagen type II (COL2A1), aggrecan (ACAN) and Sox9. These markers play a critical role in the development, formation and maintenance of cartilage tissue [52]. Similar results were also observed in other two studies. Chen *et al.* [53] found that the expression of ACAN, Sox9 and collagen II, which are markers of cartilage formation, was significantly enhanced in hydrogels containing KGN. The sustained release of KGN recruited BMSCs and promoted their proliferation and differentiation into chondrocytes. Furthermore, another study demonstrated that the incorporation of KGN into micro-particles enhanced the distribution and deposition of GAG within the MSC aggregates [54], indicating a potential role of KGN in promoting the synthesis and organization of cartilage matrix components. In our study, scaffolds specifically designed for cartilage repair were fabricated using 3D printing, incorporating KGN into a hydrogel. Building upon the findings of the aforementioned research, our study showed that KGN exhibited sustained release from the scaffold, playing a crucial role in promoting chondrogenic differentiation and facilitating cartilage regeneration.

### Evaluation of articular cartilage regeneration *in vivo*

**Figure 5.**
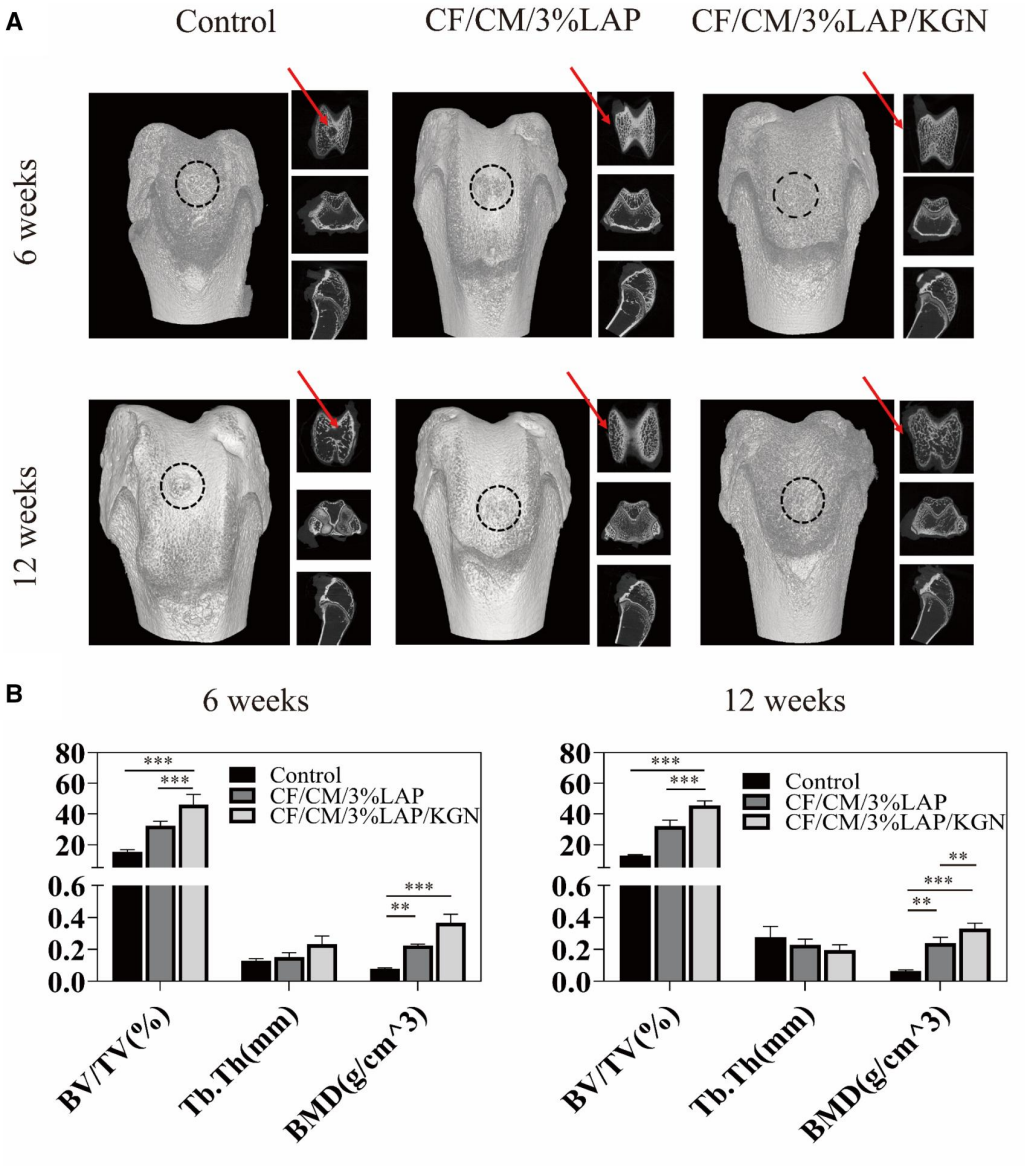
Micro-CT evaluation of repaired cartilage. (**A**) Micro-CT images showing 2D and 3D reconstruction of the repaired cartilage at 6 and 12 weeks after scaffolds implantation. (**B**) Quantitative analysis of BV/TV, Tb.Th and BMD in the defect area (*P < 0.05, **P < 0.01, ***P < 0.001).

The cartilage repair appearance diagram showed (Supplementary Figure S8) that after 12 weeks, the control group still had obvious defects, whereas the scaffold group all had varying degrees of repair, and the group loaded with KGN nanoparticles (CF/CM/3%LAP/KGN scaffolds) had essentially complete repair of the cartilage defects. Micro-CT tests in Figure 5 showed that after 6 weeks, bone volume/total volume (BV/TV), bone trabecular thickness (Tb.Th) and bone mineral density (BMD), the three indicators of subchondral bone reconstruction, were highest in the CF/CM/3%LAP/KGN scaffolds group. The BV/TV values were also considerably higher than in the other groups, suggesting that the best repair result was attained. After 12 weeks, the micro-CT analysis revealed that the CF/CM/3%LAP/KGN scaffolds group exhibited significantly higher BV/TV and BMD compared to both the control group and the scaffold group without KGN nanoparticles. However, there was no observable difference in Tb.Th between the CF/CM/3%LAP/KGN scaffolds group and the other groups.

**Figure 6.**
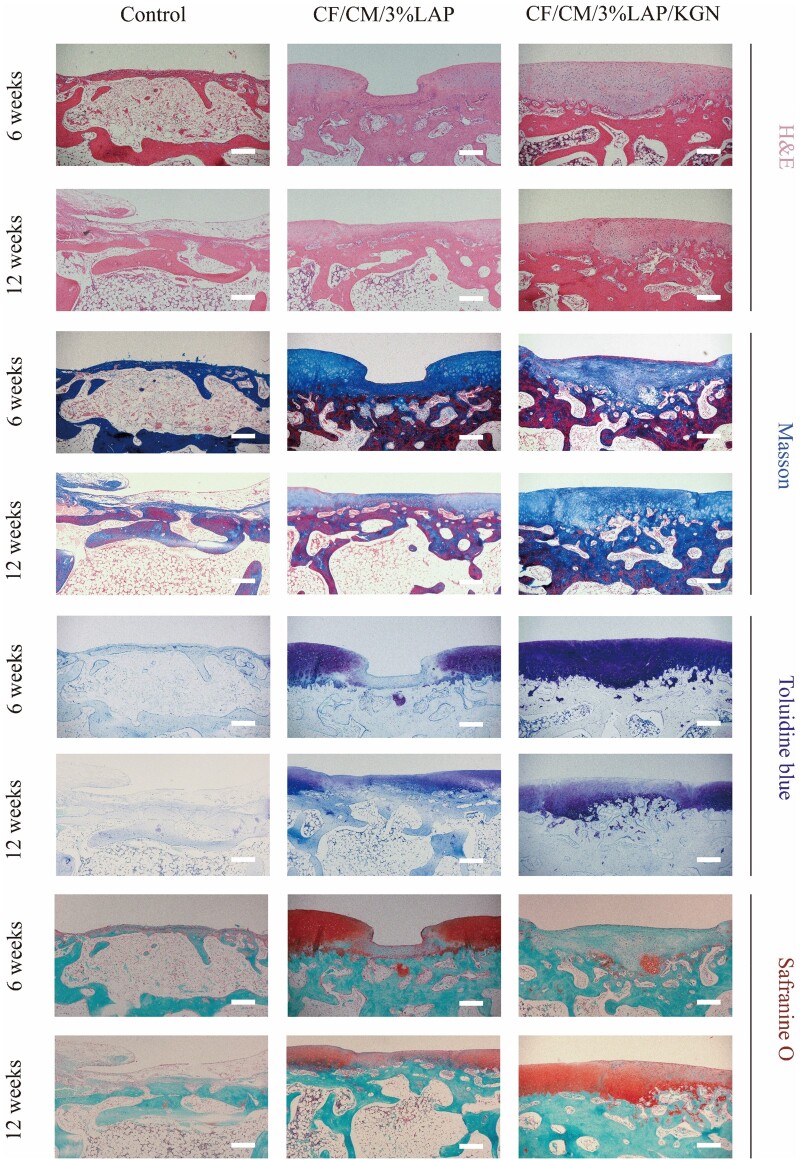
Histological evaluation of regenerated cartilage after 6 and 12 weeks postoperatively, including H&E staining, Masson staining, toluidine blue staining and Safranine-O/fast green staining. Scale bar: 250 μm.

Histologic results showed (Figure 6) that after 6 weeks, the hydrogel scaffolds were basically completely resorbed, the control group had basically no repair in the defects, the two scaffold groups had some signs of repair in the defects, and the CF/CM/3%LAP/KGN scaffolds group showed a smooth surface layer and surrounding ECM. The newborn cartilage was further detected by Masson staining, with collagen fibers in regenerated cartilage in blue, and a large number of collagen fibers were expressed in all groups except the control group. At 12 weeks after implantation, the CF/CM/3%LAP/KGN scaffolds group showed a smooth articular surface (Supplementary Figure S8). Meanwhile, the defect in the CF/CM/3%LAP/KGN scaffolds group was completely repaired by hyaline-like cartilage, a finding that was not observed in the other groups. In this group, the arrangement of cells resembled that of normal cartilage, characterized by the presence of luminal cells. Compared with the other groups, there was essentially no repair in the control group, whereas the repair tissue in the KGN-free group was irregular and abnormal. Weak toluidine blue staining and Safranine-O staining of GAGs were observed in CF/CM/3%LAP scaffolds at 6 and 12 weeks after implantation, whereas the CF/CM/3%LAP/KGN scaffolds group showed strong staining at 6 and 12 weeks after implantation (Figure 6). Moreover, a more uniform accumulation of proteoglycans and formation of neocartilaginous matrix was expressed in toluidine blue staining and Safranine-O/fast green staining in the CF/CM/3%LAP/KGN scaffolds group.

**Figure 7.**
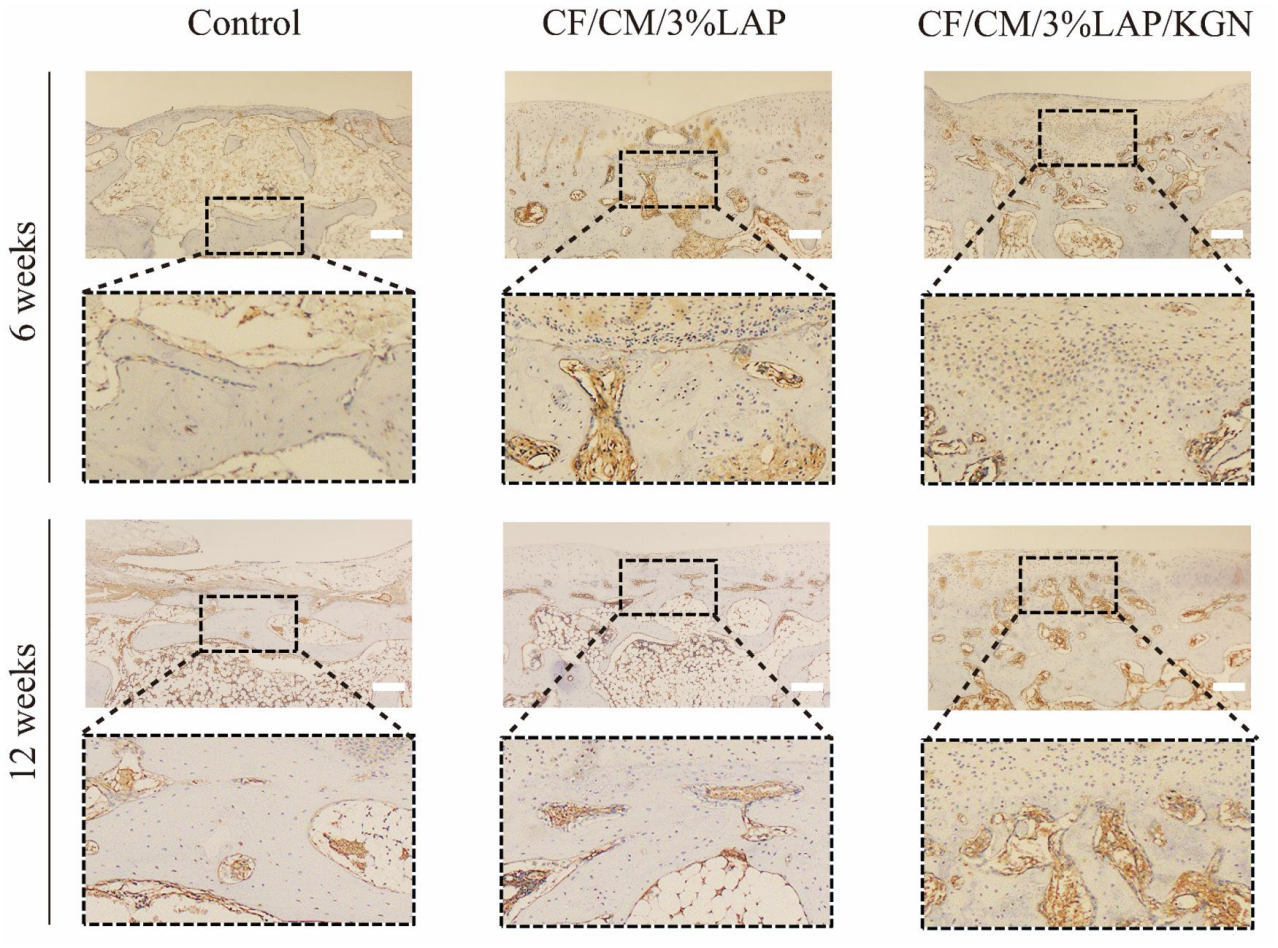
Immunohistochemical staining for collagen II in regenerated cartilage at 6 and 12 weeks postoperatively. Scale bar: 250 μm.

In addition, the expression of Col II was analyzed by immunohistochemical staining in the cartilage defect areas of each group. Results in Figure 7 showed that after 6 weeks, the detection of positive cells was low in the control and CF/CM/3%LAP scaffolds groups, but there was a high number of Col II in the CF/CM/3%LAP/KGN scaffolds group, although the defect site had not healed. After 12 weeks, it was found that the defect site in the CF/CM/3%LAP/KGN scaffolds group was closed completely, and the defect site contained Col II-expressing cells, indicating that the new cartilage was fully integrated with the surrounding tissues and that the new cartilage was hyaline cartilage. The immunohistochemical results further confirmed that the CF/CM/3%LAP/KGN scaffolds group had the best histological repair at 6 and 12 weeks.

Through *in vivo* experiments, we found that the KGN nanoparticles-loaded hydrogel scaffold set promoted cartilage regeneration and successfully integrated newborn cartilage tissue into the host cartilage structure.

## Conclusion

In conclusion, this study successfully developed a customizable, 3D-printed hydrogel scaffold for cartilage repair, where we used a combination of CF-1552, ChMA, LAP and KGN@PLGA nanoparticles to form a printable bioink, and the printed scaffolds showed sustained drug release. It was shown that the prepared CF/CM/3%LAP/KGN hydrogel scaffolds have a reticulated porous structure and good mechanical properties and can be used to fill irregular cartilage defect sites. In *in vitro* studies, the CF/CM/3%LAP/KGN scaffolds exhibited excellent biocompatibility, demonstrating outstanding antibacterial properties, and promoting the differentiation of hBMSCs into chondrocytes. *In vivo* studies, based on a rabbit cartilage defect model, the safety and ability of CF/CM/3%LAP/KGN hydrogel scaffolds to promote rapid cartilage healing were further demonstrated. The *in vivo* study’s outcomes showed this candidate material’s potential for cartilage regeneration applications, which could prove helpful in real-world uses down the road.

## Supplementary Material

rbae097_Supplementary_Data
