# Supplementary material for: 3D printing of recombinant collagen/chitosan methacrylate/nanoclay hydrogels loaded with Kartogenin nanoparticles for cartilage regeneration
Source: Regen Biomater. 2024 Aug 28;11:rbae097. doi: 10.1093/rb/rbae097 (PMC11364519; doi:10.1093/rb/rbae097)
Supplement: rbae097_Supplementary_Data [file rbae097_supplementary_data.docx]

**Supplementary Information**

3D Printing of Recombinant Collagen/Chitosan Methacrylate/ Nanoclay Hydrogels Loaded with Kartogenin Nanoparticles for Cartilage Regeneration

Wanting Zhang^a, b, c^, Kejia Shi^a, b, c^, Jianfeng Yang^a, b, c^, Wenjing Li^a, b, c^, Yang Yu^a, b, c^, Yu Mi^a, b, c^, Tianyu Yao^a, b, c^*, Pei Ma^a, b, c^*, Daidi Fan^a, b, c^*

^a^ Engineering Research Center of Western Resource Innovation Medicine Green Manufacturing, Ministry of Education, School of Chemical Engineering, Northwest University, Xi'an, 710069, China

^b^ Shaanxi Key Laboratory of Degradable Biomedical Materials and Shaanxi R&D Center of Biomaterials and Fermentation Engineering, School of Chemical Engineering, Northwest University, Xi'an, 710069, China

^c^ Biotech. & Biomed. Research Institute, Northwest University, Xi'an, 710069, China

* E-mail: t.yao@nwu.edu.cn; mapei@nwu.edu.cn; fandaidi@nwu.edu.cn

# Materials and methods

## SEM

Morphological structures of the hydrogels are probed using a Gemini SEM-500 scanning electron microscope (SEM, German Zeiss) at an accelerating voltage of 5.0 kV. Freeze-dried hydrogels are microtomed into 2mm thin sections under liquid nitrogen,subsquently sputtered with gold for 80s before imaging. For the CF/CM, CF/CM/3%LAP and CF/CM/4%LAP hydrogels, 30 pore sizes were randomly measured from the corresponding SEM images of each hydrogel by image J software and histograms of the pore size distribution were generated using origin software.

## The charge distribution

The charge distribution of the hydrogels containing the different component materials was obtained by ZETA Potential Analyser (Anton Paar, Litesizer). Five-fold dilution of CF/CM, CF/CM/3%LAP, CF/CM/3%LAP/KGN, CF/CM/4%LAP and CF/CM/4%LAP/KGN hydrogels with deionised water was used to prevent samples from being too concentrated for measurement (n=3).

## Dissolution rate

The initial weight of the scaffolds was weighed. The scaffolds were then placed in PBS (pH = 7.4, 37°C), removed at predetermined time points and weighed. Its dissolution rate was calculated.

## In vitro degradation of scaffolds

The lyophilized scaffold samples were sealed in PBS solution and placed in a constant temperature incubation shaker (37 °C, 150 rpm). The scaffolds were removed at different time points and weighed.

## Hematocompatibility assay

Fresh blood was collected from the marginal vein of the rabbit ear, and the erythrocytes were centrifuged (5000 rpm, 5 min) and rinsed twice with physiological saline, and then diluted into a 5% (v/v) solution. Saline was added to the different stent samples according to the standard of 0.1 g/mL, and the extracts were added to the erythrocyte solution after immersion at 37°C for 24 h. Distilled water and saline were added to the control group, respectively. After 1 h of incubation, the absorbance of the supernatant was detected at 540 nm with ELISA(United States, Biotek).

## Cell migration assay

The hBMSCs were inoculated in 6-well plates at a density of 1.2×10^6^ cells/well, and after the cells were completely covered the bottom of the 6-well plates, the cells were vertically scribed using a 10 μL sterile pipette gun tip, and the old culture solution was aspirated and washed twice with PBS, after which the cells were treated with low-serum DMEM culture solution(Gibco) and the scaffolds, respectively, to obtain the control and experimental groups, which were cultured for 24 h. The control and experimental groups were inoculated and photographed by using an inverted microscope (Japan, Nikon), three randomly selected areas were photographed and quantified by Image J software.

## Antibacterial activity

The hydrogel scaffolds were co-cultured with bacterial suspensions of Hepatococcus coli and Staphylococcus aureus for 24 h. 100 μl of each was aspirated and spread on agar plates. Colonies were counted after 24h of incubation at 37°C to determine the respective antibacterial activity.

## hBMSCs seeded on 3D scaffold

The scaffolds implanted with hBMSCs were incubated for 24 h, rinsed with PBS, fixed using 4% paraformaldehyde for 20 min, rinsed with PBS and then treated with 0.1% Triton-100 for 20 min. Lastly, the cells on the scaffolds were stained with Vari Fluor 555-Phalloidin for 30 min followed by 4’, 6-diamidino-2-phenylindole (DAPI) for another 15 min.


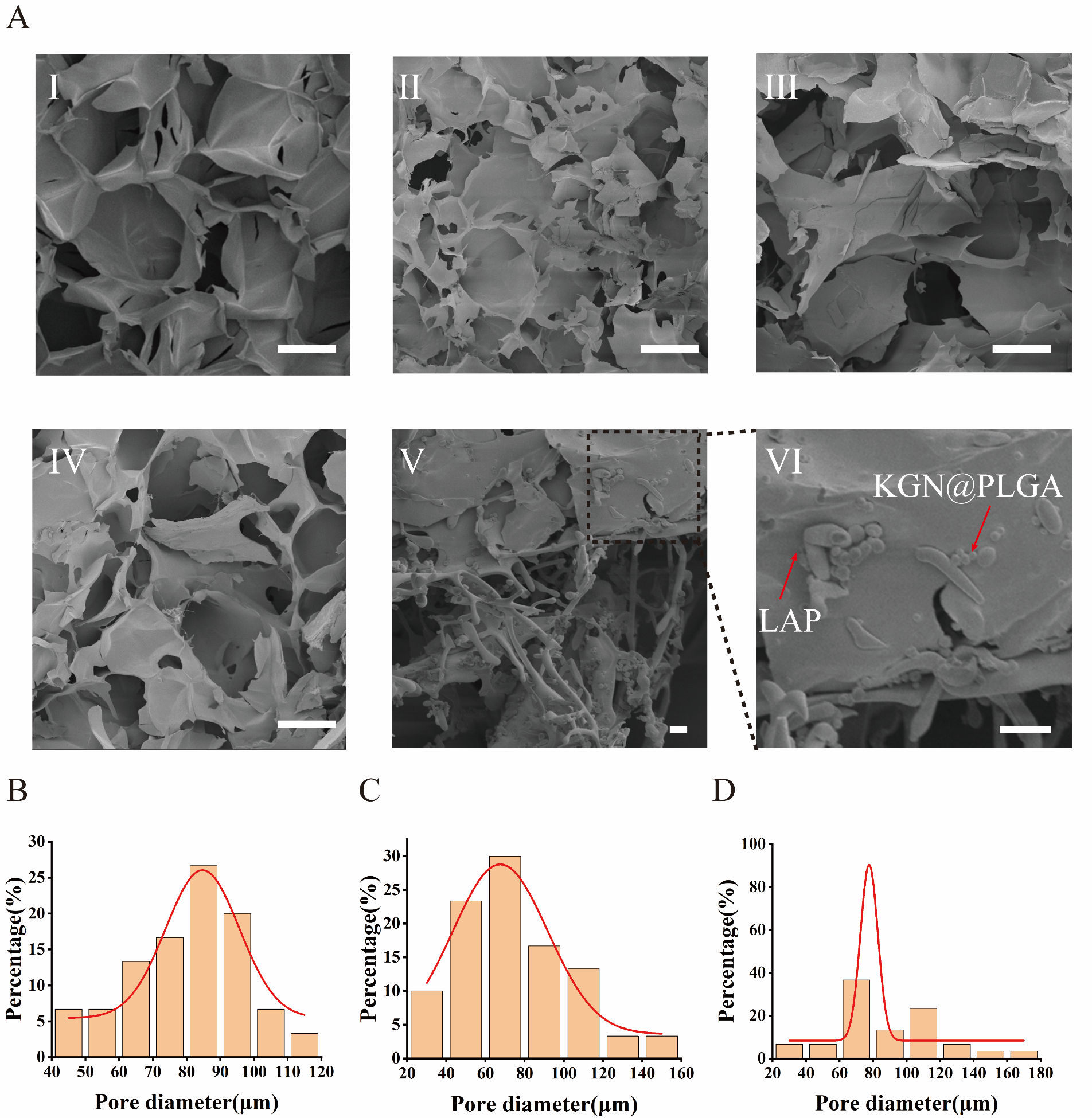


Figure S1. The corresponding SEM images of the hydrogels after printing. (A) (Ⅰ) CF/CM （Ⅱ）CF/CM/3%LAP （Ⅲ） CF/CM/4%LAP （Ⅳ-Ⅵ） CF/CM/3%LAP/KGN. (Ⅰ-Ⅳ) scale bars: 100μm. (Ⅴ-Ⅵ) scale bars: 2μm. (B) Pore size distribution histogram of CF/CM hydrogel. (C) Pore size distribution histogram of CF/CM/3%LAP hydrogel. (D) Pore size distribution histogram of CF/CM/4%LAP hydrogel.


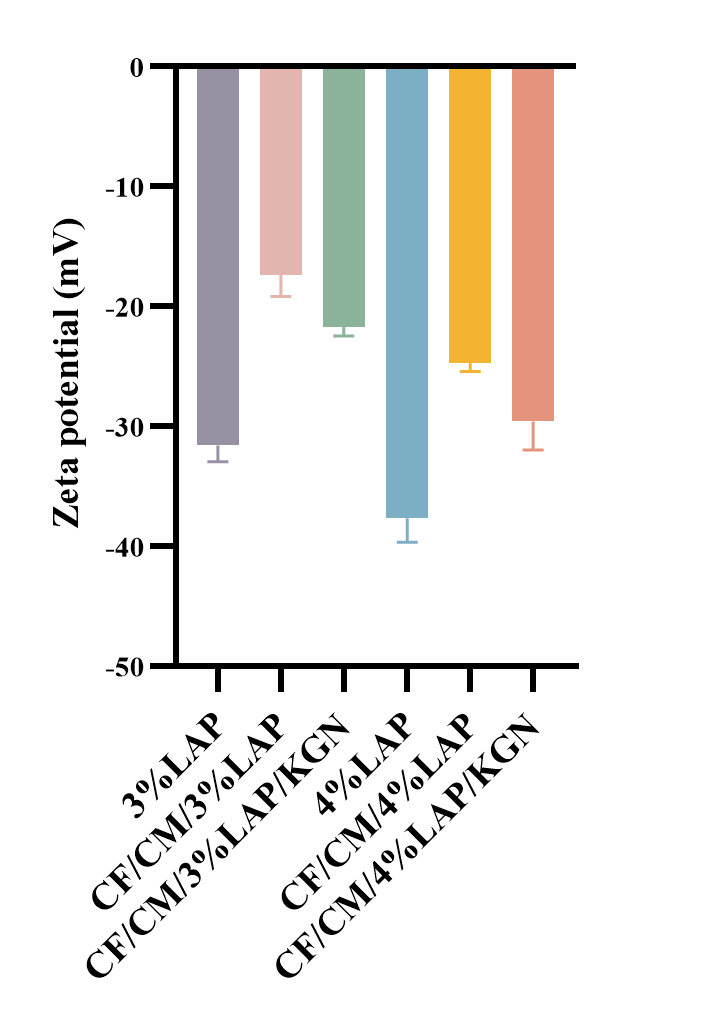


Figure S2. The zeta potentials of materials containing different compositions.


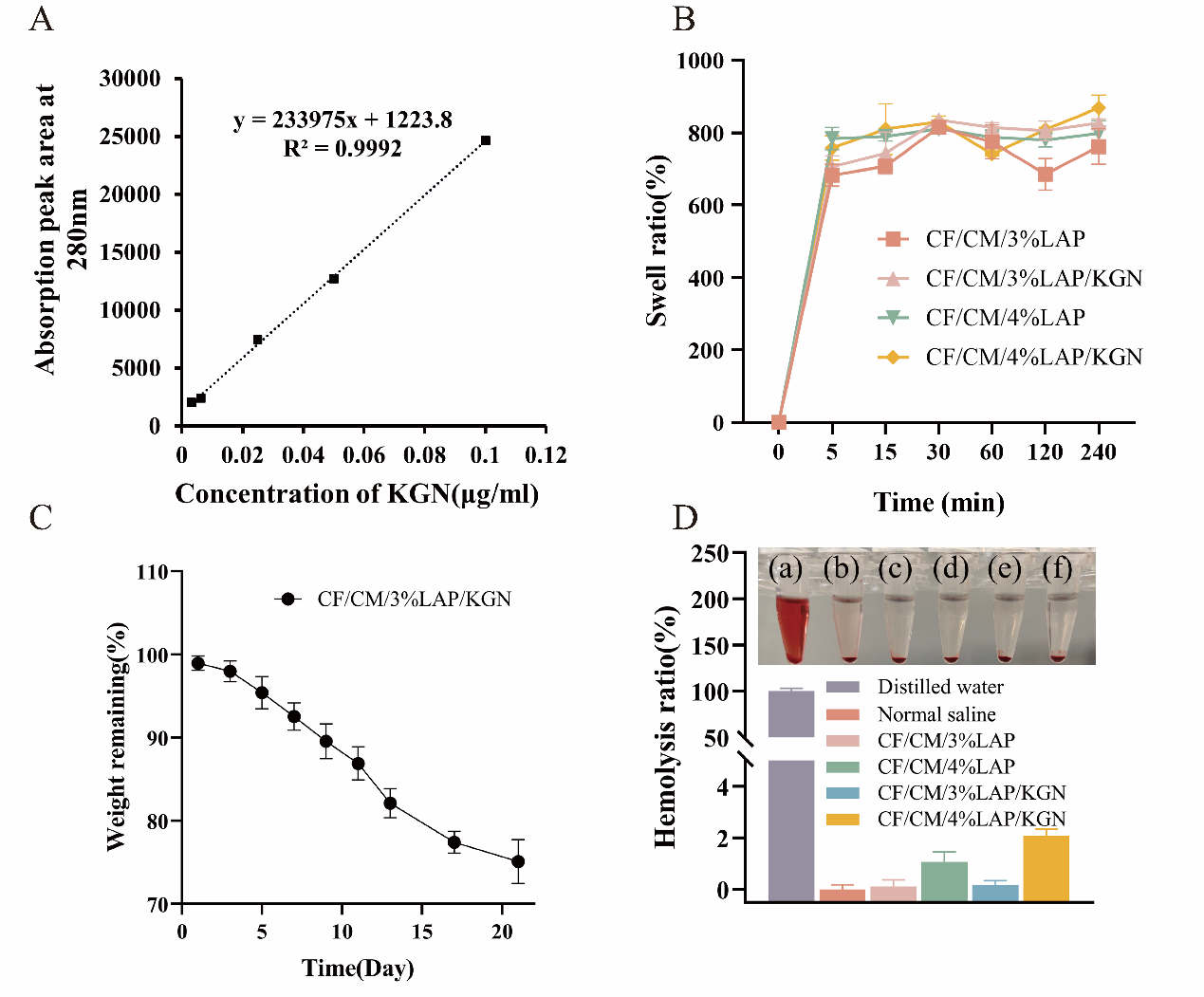


Figure S3. (A) Standard curve of kartogenin (KGN). (B) Dissolution rate of scaffolds. (C) Degradation of CF/CM/3%LAP/KGN scaffold in PBS. (D) Photographs of blood erythrocytes and hemolysis rate in stent hemolysis experiments.


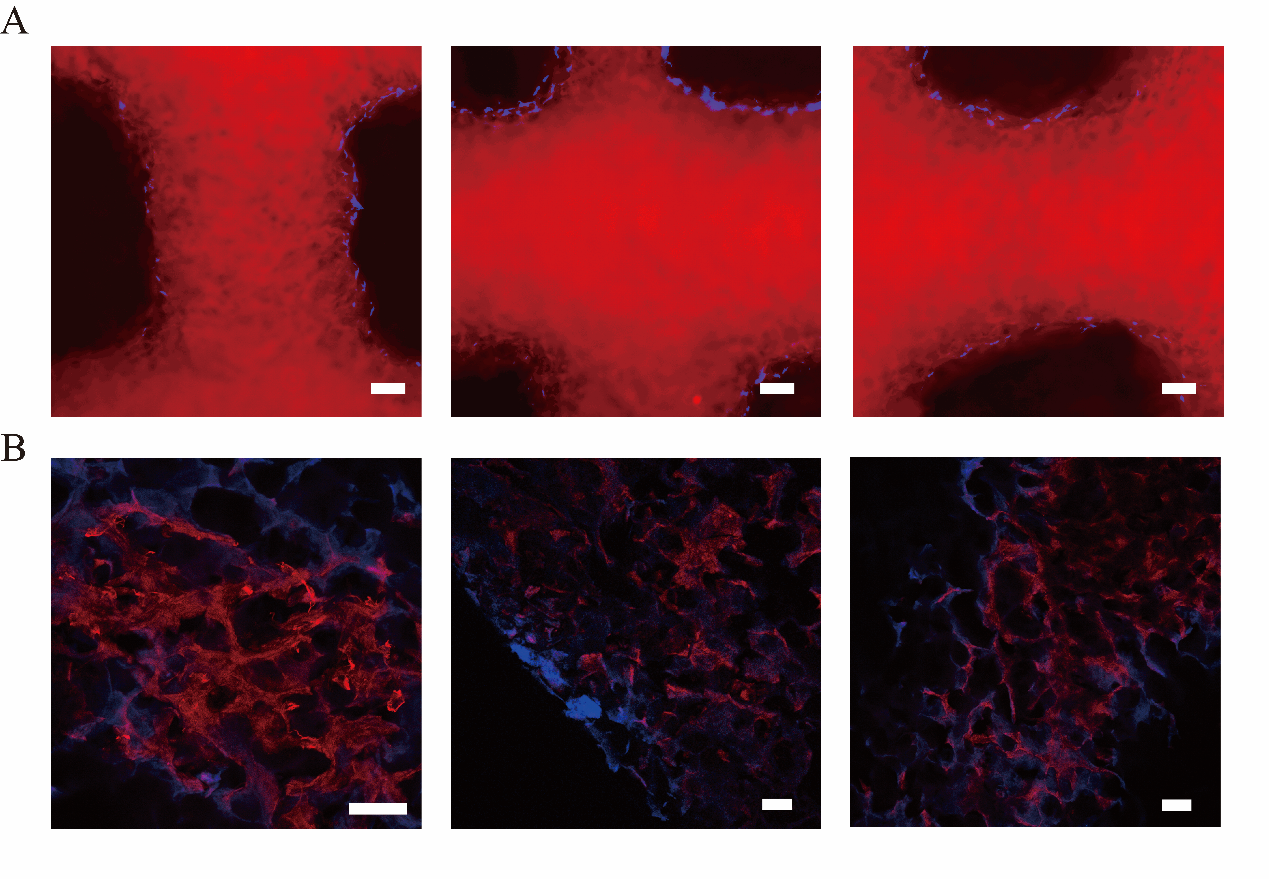


Figure S4.(A) Fluorescence microscopy and (B) laser confocal microscopy photographs of hBMSCs co-cultured with the scaffolds, stained by Vari Fluor 555-Phalloidin (red) and DAPI (blue). Scale bar: 100 μm.


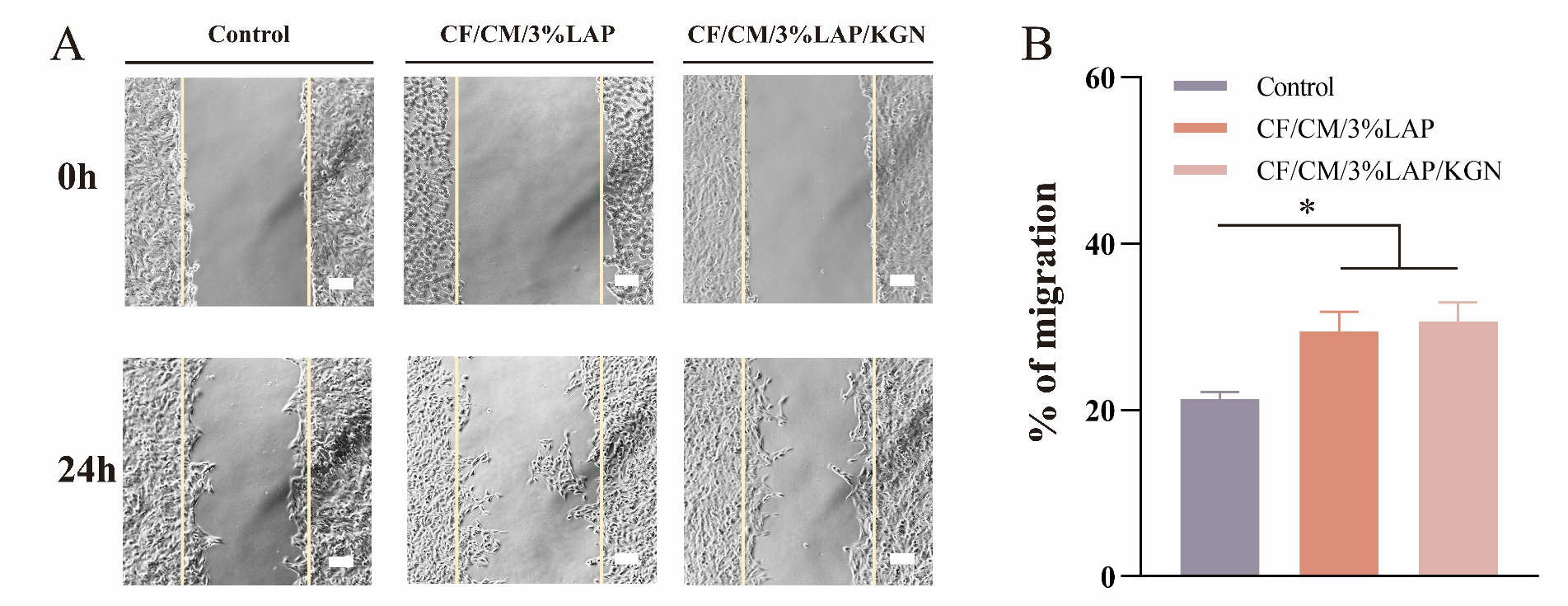


Figure S5. (A) Scratch test photographs and (B) cell mobility calculations of hBMSCs induced by different scaffolds. Scale bar: 200 μm. (**P* < 0.05)


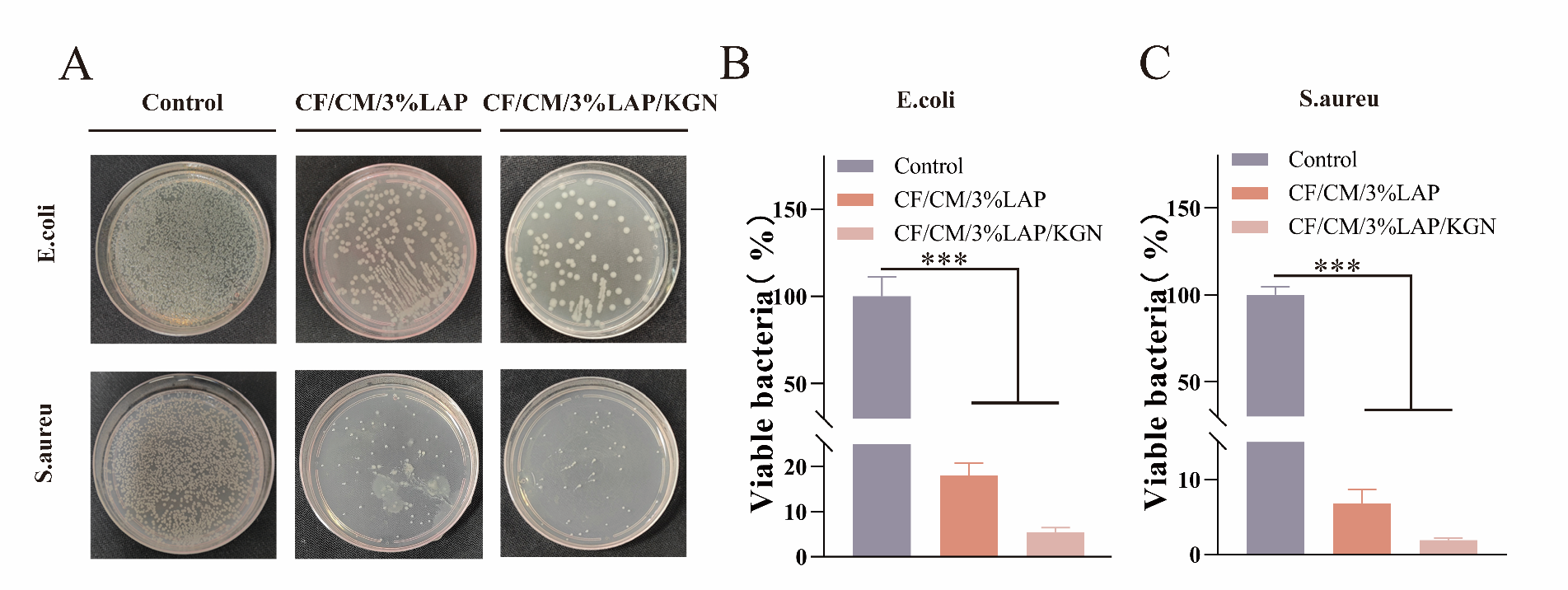


Figure S6. (A) Image of viable bacterial CFUs Plot of plate-coated bacterial counts for testing the antimicrobial properties of the stent. (B, C) Bacteriostatic rate of E. coli and S. aureus. (****P* < 0.001)


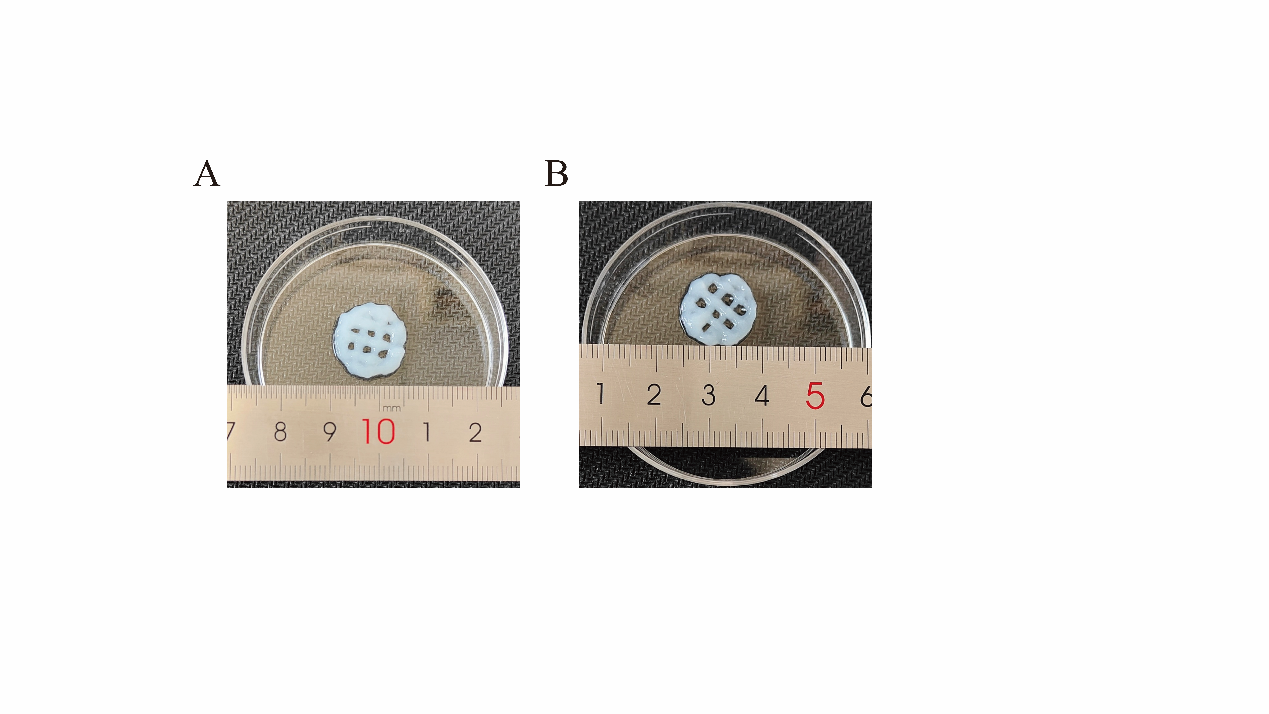


Figure S7. Schematic diagram of scaffolds used for cellular experiments(A) CF/CM/3%LAP. (B) CF/CM/3%LAP/KGN.


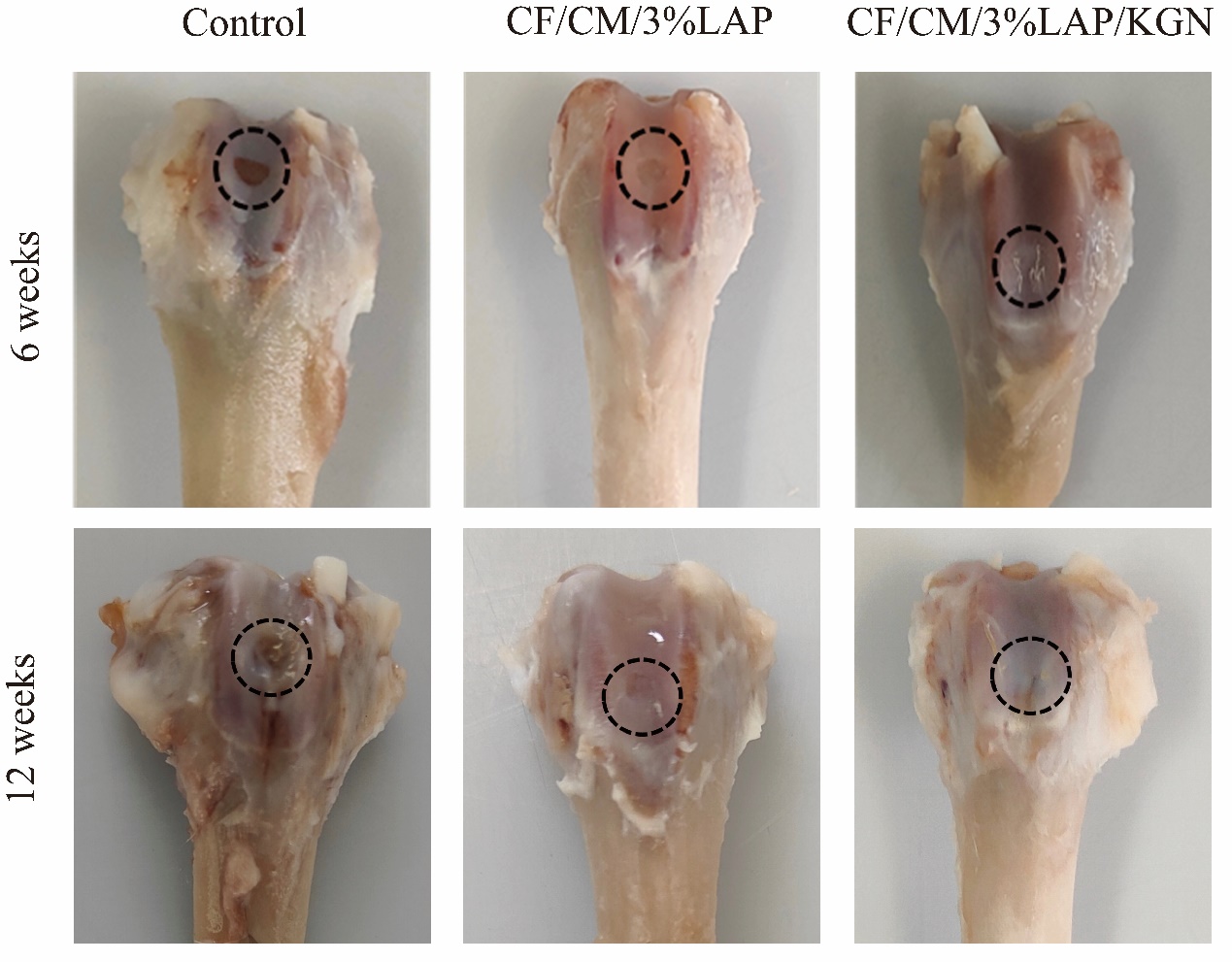


Figure S8. Appearance of repaired tissue at 6 and 12 weeks postoperatively.
